# The Gut–Brain Axis in Metabolic Syndrome: Emerging Mechanisms and Perspectives in Personalized Medicine

**DOI:** 10.3390/ijms27156622

**Published:** 2026-07-24

**Authors:** Lucia Maria Procopciuc, Adriana Corina Hangan, Roxana Liana Lucaciu

**Affiliations:** 1 Department of Medical Biochemistry, Faculty of Medicine, “Iuliu Hațieganu” University of Medicine and Pharmacy, 400349 Cluj-Napoca, Romania; 2 Department of Inorganic Chemistry, Faculty of Pharmacy, “Iuliu Hațieganu” University of Medicine and Pharmacy, 400012 Cluj-Napoca, Romania; 3 Department of Pharmaceutical Biochemistry and Clinical Laboratory, Faculty of Pharmacy, “Iuliu Hațieganu” University of Medicine and Pharmacy, 400349 Cluj-Napoca, Romania; liana.lucaciu@umfcluj.ro

**Keywords:** metabolic syndrome, obesity, insulin resistance, dyslipidemia, hypertension, impaired glucose metabolism, gut–brain axis

## Abstract

Metabolic syndrome (MetS) is a multifactorial disorder characterized by central obesity, insulin resistance, dyslipidemia, hypertension, and impaired glucose metabolism, significantly increasing the risk of type 2 diabetes and cardiovascular disease. Recent evidence highlights the important role of the gut–brain axis in the pathogenesis of MetS through complex interactions between the gut microbiota, immune system, endocrine signaling, and host genetics. This narrative review provides an integrative overview of the mechanisms linking dysbiosis to metabolic dysfunction, with particular emphasis on gut microbiota alterations, intestinal permeability, chronic low-grade inflammation, and microbial metabolites such as short-chain fatty acids and lipopolysaccharides. The review also discusses the neural, endocrine, and immune pathways involved in gut–brain communication, including the role of gut-derived neurotransmitters in metabolic regulation. In addition, the contribution of host genetic susceptibility and epigenetic regulation is explored, highlighting how gene–microbiome interactions influence individual metabolic responses and disease risk. Recent advances in multi-omics technologies and precision medicine suggest that personalized approaches targeting both microbial and genetic factors may improve prevention and treatment strategies for MetS. Furthermore, microbiota-targeted interventions, including dietary modifications, probiotics, prebiotics, and fecal microbiota transplantation, are discussed as emerging therapeutic perspectives. Overall, this review emphasizes the importance of considering MetS as a systemic disorder driven by interconnected biological networks involving microbiota, metabolism, immunity, and genetics.

## 1. Introduction

Metabolic syndrome (MetS) is a complex cardiometabolic disorder characterized by the clustering of central obesity, dyslipidemia, elevated blood pressure, and impaired glucose regulation. According to the harmonized definition, MetS is diagnosed when at least three of five criteria are present: increased waist circumference, elevated triglycerides, reduced HDL-C, elevated blood pressure, and elevated fasting plasma glucose [1].

Although clinically defined by these measurable components, MetS reflects a broader systemic disturbance involving impaired metabolic homeostasis and multiple interacting biological pathways. MetS is clinically relevant because its components act synergistically to increase the risk of atherosclerotic cardiovascular disease, type 2 diabetes mellitus, NAFLD/MASLD, PCOS, obstructive sleep apnea, and other obesity-related complications [2].

Its global burden has increased substantially in parallel with obesity, sedentary lifestyles, urbanization, and dietary changes. Prevalence estimates vary according to diagnostic criteria, age, sex, socioeconomic status, and geographic region, with global and European data indicating that MetS represents a major and growing public health problem [3,4,5].

The major pathophysiological mechanisms underlying MetS include insulin resistance, visceral adiposity, dyslipidemia, chronic low-grade inflammation, mitochondrial dysfunction, oxidative stress, and neuroendocrine dysregulation. These mechanisms do not act independently; instead, they reinforce each other through interconnected metabolic and inflammatory pathways. For example, visceral adiposity promotes the release of free fatty acids and pro-inflammatory cytokines, which impair insulin signaling, alter lipid metabolism, and contribute to endothelial dysfunction. Thus, MetS should be understood not only as a cluster of clinical abnormalities but also as a systemic network disorder involving metabolic, immune, endocrine, and environmental determinants. Within this network, the gut–brain axis (GBA) has emerged as an important regulatory system linking the gastrointestinal tract, gut microbiota, enteric nervous system, vagus nerve, immune system, endocrine signaling, and central nervous system [6,7,8,9]. In MetS, GBA dysregulation may influence appetite regulation, energy expenditure, glucose homeostasis, stress responses, inflammation, autonomic balance, and metabolic flexibility. Gut-to-brain and brain-to-gut communication occurs through neural signaling via the vagus nerve and enteric nervous system, endocrine pathways involving the hypothalamic–pituitary–adrenal axis, immune mediators such as cytokines, and metabolic signals derived from microbial activity [10,11].

Microbial metabolites represent key biochemical mediators connecting diet, microbiota composition, gut barrier function, liver metabolism, gut–brain signaling, and cardiometabolic dysfunction. The gut microbiota produces bioactive molecules such as short-chain fatty acids (SCFAs), bile acid derivatives, lipopolysaccharide (LPS), trimethylamine N-oxide (TMAO)-related precursors, tryptophan metabolites, and neuroactive compounds, including serotonin and gamma-aminobutyric acid (GABA) [12,13]. More specifically, MetS-related gut–brain communication appears to involve defined microbial taxa and metabolites, including SCFA-producing bacteria such as *Faecalibacterium prausnitzii* and *Roseburia* spp., mucus-associated *Akkermansia muciniphila*, LPS-producing Gram-negative bacteria, bile acid-transforming bacteria, TMA-producing taxa, and tryptophan-metabolizing microorganisms. These microbial groups may influence metabolic and neuroendocrine regulation through FFAR2/3, GPR109A, FXR/TGR5, TLR4/NF-κB, AhR, enteroendocrine hormone secretion, vagal signaling, and HPA axis-related pathways. These molecules may influence intestinal permeability, immune activation, enteroendocrine hormone secretion, appetite regulation, insulin sensitivity, lipid metabolism, and systemic inflammation. Therefore, dysbiosis may contribute to MetS not only through altered microbial composition, but also through changes in microbial metabolic output.

Recent evidence also suggests that MetS arises from interactions between host genetic background, epigenetic regulation, and the gut microbiome. Genetic variants involved in immunity, metabolism, intestinal barrier function, and microbial colonization may influence gut microbiota composition, while microbiota-derived metabolites may regulate host gene expression through epigenetic and metabolic mechanisms. These bidirectional interactions contribute to interindividual variability in metabolic responses and may partly explain differences in susceptibility to obesity, insulin resistance, and related metabolic disorders. Consequently, gene–microbiome interactions have become increasingly relevant for precision medicine approaches in MetS [14,15,16,17,18,19].

Although recent reviews have addressed gut microbiota, GBA signaling, inflammation, or microbiota-targeted interventions in obesity and metabolic disorders, fewer have integrated these mechanisms with host genetic susceptibility, epigenetic regulation, microbial metabolites, and personalized medicine in the specific context of MetS. Therefore, this review aims to provide an integrative synthesis of the gut microbiota–GBA–host genetics network in MetS, emphasizing dysbiosis, intestinal barrier dysfunction, microbial metabolites, chronic low-grade inflammation, neuroendocrine signaling, gene–microbiome interactions, epigenetic regulation, and personalized therapeutic perspectives. To align with this aim, the review is structured around specific microbiota-derived molecular routes linking gut dysbiosis with MetS, including SCFA–FFAR2/3/GPR109A signaling, LPS–TLR4/NF-κB-mediated inflammation, bile acid–FXR/TGR5 signaling, TMA–TMAO metabolism, tryptophan–indole–AhR signaling, enteroendocrine GLP-1/PYY secretion, vagal and HPA axis signaling, and gene–microbiome–epigenetic interactions.

The novelty of this review lies in its integrative approach, linking gut dysbiosis, gut–brain axis signaling, host genetic and epigenetic regulation, and personalized medicine into a unified framework for understanding MetS pathogenesis and future management.

## 2. Methodology

This narrative review was conducted to provide a comprehensive synthesis of current evidence regarding the GBA and its role in the pathogenesis of MetS. The review includes a broad range of study designs, such as observational studies (cross-sectional, case–control, and cohort studies) and experimental research (in vitro and in vivo studies), as well as recent systematic reviews and meta-analyses. This approach allows for the integration of mechanistic insights with clinical and epidemiological findings.

A structured literature search was performed using major electronic databases, including PubMed/MEDLINE, Scopus, and Web of Science. Additional relevant articles were identified through manual screening of reference lists of selected publications. The search strategy combined keywords and Medical Subject Headings (MeSH) terms related to “gut–brain axis,” “microbiome,” “metabolic syndrome,” “insulin resistance,” and “genetics,” with a focus on studies published in the last 5–10 years to ensure the inclusion of recent advances.

Studies were included if they addressed the interaction between the gut microbiome, central nervous system, and metabolic processes, particularly in relation to metabolic syndrome or its individual components.

## 3. Metabolic Syndrome—Pathophysiological Mechanisms

### 3.1. Insulin Resistance

Insulin resistance represents the central pathophysiological mechanism underlying MetS, playing a key role in the development and interconnection of its major components, hyperglycemia, dyslipidemia, hypertension, and abdominal obesity. It is defined as a diminished biological response of peripheral tissues, primarily skeletal muscle, liver, and adipose tissue, to the action of insulin, leading to impaired glucose uptake and compensatory hyperinsulinemia [20,21].

At the molecular level, insulin signaling is a highly coordinated process that enables cells to regulate glucose homeostasis in response to circulating insulin. Under normal physiological conditions, insulin binds to the insulin receptor (IR), a transmembrane receptor with intrinsic tyrosine kinase activity. This binding induces receptor autophosphorylation on specific tyrosine residues, which in turn promotes the recruitment and phosphorylation of insulin receptor substrates, primarily Insulin Receptor Substrate-1 (IRS-1) and IRS-2 [22,23].

Once phosphorylated on tyrosine residues, IRS-1 serves as a docking platform for multiple signaling molecules, most importantly phosphatidylinositol-3-kinase (PI3K). Activation of PI3K leads to the conversion of phosphatidylinositol-4,5-bisphosphate (PIP2) into phosphatidylinositol-3,4,5-trisphosphate (PIP3), a key second messenger. PIP3 facilitates the recruitment and activation of protein kinase B (Akt), which represents a central node in the insulin signaling cascade. Activated Akt mediates several metabolic effects, including the translocation of GLUT4-containing vesicles from intracellular compartments to the plasma membrane, thereby enabling glucose uptake, particularly in skeletal muscle and adipose tissue [22].

In addition to promoting glucose uptake, Akt regulates other metabolic pathways. It suppresses hepatic gluconeogenesis by inhibiting transcription factors such as Forkhead Box protein O1 (FOXO1) and enhances glycogen synthesis through inhibition of glycogen synthase kinase-3 (GSK-3). Through these coordinated actions, insulin signaling maintains systemic glucose balance by both increasing peripheral glucose utilization and decreasing hepatic glucose production [21,22].

In MetS, this signaling pathway becomes significantly impaired. A central defect involves the abnormal phosphorylation of IRS-1 on serine residues rather than tyrosine residues. This serine phosphorylation reduces the ability of IRS-1 to interact with the insulin receptor and to activate downstream signaling molecules such as PI3K. As a result, the entire signaling cascade is attenuated, leading to decreased Akt activation and impaired glucose transport [24,25].

This inhibitory phosphorylation is driven by multiple factors associated with metabolic syndrome, including chronic low-grade inflammation. Adipose tissue—especially visceral fat—acts as an active endocrine organ, secreting pro-inflammatory cytokines, such as tumor necrosis factor α (TNF-α) and interleukin-6 (IL-6), and elevated levels of free fatty acids (FFAs). These cytokines activate intracellular inflammatory pathways, including nuclear factor kappa B (NF-κB) and c-Jun N-terminal kinase (JNK) and the mechanistic target of rapamycin/ribosomal protein S6 kinase (mTOR/S6K) pathway, all of which interfere with insulin signaling and promote insulin resistance and contribute to the dysfunctional modification of IRS-1 [23,24,25,26]. Concurrently, levels of adiponectin, an anti-inflammatory and insulin-sensitizing adipokine, are reduced, further exacerbating metabolic dysfunction [27].

Consequently, reduced PI3K/Akt signaling leads to impaired translocation of GLUT4 transporters to the cell membrane, significantly decreasing glucose uptake in skeletal muscle and adipose tissue. At the same time, in the liver, insulin fails to adequately suppress gluconeogenesis, resulting in increased hepatic glucose output and contributing to fasting hyperglycemia [22,26].

Lipotoxicity is an important contributor to insulin resistance. In obesity, increased adipose tissue lipolysis elevates circulating FFAs, which accumulate ectopically in the liver and skeletal muscle. Their conversion into lipid intermediates such as diacylglycerols and ceramides activates protein kinase C isoforms, impairs IRS signaling, and reduces insulin sensitivity, thereby linking lipid overload to defective glucose metabolism [25,26].

Mitochondrial dysfunction also plays an important role in the pathogenesis of insulin resistance. A decreased capacity for fatty acid oxidation, along with increased oxidative stress, leads to the accumulation of metabolic intermediates that interfere with cellular energy homeostasis and insulin sensitivity. Thus, impaired mitochondrial function is closely associated with the metabolic disturbances observed in MetS [28].

At the organ level, insulin resistance manifests differently across tissues. In the liver, it results in increased gluconeogenesis and excessive hepatic glucose output, contributing to fasting hyperglycemia. It also promotes hepatic lipid synthesis, leading to hypertriglyceridemia. In skeletal muscle, insulin resistance reduces glucose uptake and glycogen synthesis, significantly contributing to postprandial hyperglycemia. In adipose tissue, it enhances lipolysis and the release of FFAs, perpetuating a vicious metabolic cycle [21,25].

Recent studies have identified gut microbiota dysbiosis as an additional factor contributing to insulin resistance. Through its effects on intestinal barrier integrity, inflammatory signaling, and microbial metabolite production, the microbiota may influence glucose homeostasis and insulin sensitivity, as discussed in Section 4.1 [29,30]. Insulin resistance at the molecular level arises from a convergence of inflammatory signaling, lipid-induced toxicity, and stress-related kinase activation, all of which impair the function of IRS-1 and disrupt the PI3K/Akt pathway. This results in reduced glucose uptake, increased hepatic glucose production, and ultimately the metabolic disturbances characteristic of insulin resistance and MetS.

### 3.2. Dyslipidemia

Dyslipidemia is a fundamental component of MetS and a major driver of atherosclerotic cardiovascular disease. It is characterized by a distinct lipid profile known as atherogenic dyslipidemia, which includes elevated triglycerides, reduced HDL-C, increased apolipoprotein B (ApoB), and the predominance of small, low-density lipoprotein cholesterol (LDL-C) particles [31,32]. These abnormalities are closely linked to insulin resistance and adipose tissue dysfunction, which together disrupt normal lipid metabolism.

A central mechanism underlying dyslipidemia in MetS is increased hepatic production of very low-density lipoproteins (VLDL). In insulin-resistant states, the inhibitory effect of insulin on adipose tissue lipolysis is diminished, leading to excessive release of FFAs into the circulation. These FFAs are taken up by the liver and re-esterified into triglycerides, which are subsequently packaged into VLDL particles and released into the bloodstream, resulting in hypertriglyceridemia [2,33]. This process is further amplified by the impaired ability of insulin to suppress hepatic lipid synthesis and gluconeogenesis.

In addition to increased production, there is also reduced clearance of triglyceride-rich lipoproteins. Insulin resistance is associated with decreased activity of lipoprotein lipase (LPL), the key enzyme responsible for hydrolyzing triglycerides in circulating lipoproteins. Moreover, increased levels of apolipoprotein C-III (ApoC-III), an inhibitor of LPL, further impair the catabolism of VLDL and chylomicrons. This leads to the accumulation of triglyceride-rich remnants, which are highly atherogenic and contribute to endothelial dysfunction [32]. Another hallmark of dyslipidemia in metabolic syndrome is low HDL-C levels and impaired HDL function. Enhanced activity of cholesteryl ester transfer protein (CETP) promotes the exchange of triglycerides from VLDL to HDL-C in exchange for cholesteryl esters. As a result, HDL-C particles become enriched in triglycerides and are more susceptible to hydrolysis by hepatic lipase, leading to the formation of smaller, dysfunctional HDL-C particles that are rapidly cleared from circulation. This reduces the efficiency of reverse cholesterol transport, a key anti-atherogenic mechanism [33].

LDL particles are also qualitatively altered. Although total LDL-C levels may be normal or only mildly elevated, there is a shift toward small dense LDL-C particles, which are more atherogenic due to their increased susceptibility to oxidation, greater penetration into the arterial wall, and prolonged plasma residence time. These particles play a critical role in the initiation and progression of atherosclerosis [31,32].

Adipose tissue dysfunction further amplifies dyslipidemia through increased FFA release, chronic inflammation, and impaired lipoprotein metabolism [2,34].

Excess lipid accumulation in tissues leads to mitochondrial overload, increased production of reactive oxygen species (ROS), and impairment of fatty acid oxidation. These changes not only exacerbate lipid abnormalities but also contribute to endothelial dysfunction and cardiovascular risk [31].

Additionally, emerging research suggests that the gut microbiota influences lipid metabolism through modulation of bile acid signaling, lipid absorption, and systemic inflammation. Dysbiosis has been associated with altered lipid profiles and may contribute to the development of dyslipidemia in MetS through complex metabolic and immune pathways [33].

From a clinical perspective, recent guidelines emphasize that dyslipidemia in MetS should not be assessed solely by LDL-C levels. Instead, a broader evaluation including non-HDL-C, ApoB, triglyceride-rich lipoproteins, and lipoprotein(a) is recommended to better estimate cardiovascular risk [35]. This reflects an evolving understanding that multiple atherogenic lipoproteins contribute to disease progression.

### 3.3. Visceral Adiposity

Visceral obesity is a central component of MetS and is considered a stronger predictor of cardiometabolic risk than overall obesity measured by body mass index (BMI). It is defined as the excessive accumulation of adipose tissue within the abdominal cavity, surrounding internal organs such as the liver, pancreas, intestines, and kidneys [31,36]. Unlike subcutaneous adipose tissue, visceral adipose tissue (VAT) is highly metabolically active and functions as an endocrine and immunologically active organ that plays a crucial role in systemic metabolic regulation and inflammation [31,37].

At the cellular level, visceral adiposity is characterized by both adipocyte hypertrophy and hyperplasia. During chronic positive energy balance, adipocytes enlarge beyond their physiological storage capacity, resulting in local hypoxia, endoplasmic reticulum stress, mitochondrial dysfunction, and activation of inflammatory pathways [38,39]. Hypoxia-induced activation of hypoxia-inducible factor-1α (HIF-1α) promotes extracellular matrix remodeling, fibrosis, and adipocyte dysfunction, reducing the capacity of adipose tissue to safely store lipids and favoring ectopic fat deposition in other organs.

Expansion of VAT is accompanied by immune cell infiltration and increased production of pro-inflammatory mediators, contributing to systemic inflammation and metabolic dysfunction. The inflammatory mechanisms linking adipose tissue dysfunction to MetS are discussed in detail in a subsequent section [33,37,40,41]. These changes contribute to systemic inflammation, endothelial dysfunction, and impaired insulin signaling.

A key pathophysiological feature of visceral obesity is its close association with insulin resistance. Visceral adipose tissue exhibits increased lipolytic activity and enhanced responsiveness to catecholamine stimulation, resulting in excessive release of FFAs into the portal circulation [21]. According to the “portal hypothesis,” these FFAs are delivered directly to the liver, where they stimulate triglyceride synthesis, hepatic steatosis, gluconeogenesis, and increased production of VLDL, thereby contributing to dyslipidemia, hyperglycemia, and systemic insulin resistance [21,42]. In addition, intracellular accumulation of lipid intermediates such as DAGs and ceramides activates protein kinase C (PKC) isoforms, impairing IRS signaling and reducing activation of the PI3K/Akt pathway [25].

Visceral adiposity also contributes to metabolic dysfunction through mitochondrial impairment and oxidative stress. Excess nutrient availability and lipid overload increase mitochondrial ROS production, resulting in oxidative stress and impaired oxidative phosphorylation [28,32]. Elevated ROS levels activate inflammatory pathways such as nuclear factor kappa B (NF-κB) and JNK, further exacerbating insulin resistance and metabolic dysregulation [43].

Beyond its local effects, VAT influences multiple metabolic organs through the release of FFAs, adipokines, and inflammatory mediators. These factors promote hepatic steatosis, dyslipidemia, β-cell dysfunction, and systemic insulin resistance, thereby contributing to the progression of MetS [21,31,34,44].

Recent evidence also highlights the interaction between visceral adiposity and gut microbiota dysbiosis. Alterations in gut microbial composition may increase intestinal permeability and facilitate translocation of bacterial lipopolysaccharide (LPS) into the circulation, a phenomenon known as metabolic endotoxemia. Activation of toll-like receptor-4 (TLR4)-mediated inflammatory pathways amplifies adipose tissue inflammation and insulin resistance, creating a vicious cycle between visceral obesity, inflammation, and metabolic dysfunction [29].

Beyond its metabolic effects, visceral adiposity exerts important cardiovascular consequences. Increased secretion of pro-inflammatory cytokines, adipokines, and pro-thrombotic mediators contributes to endothelial dysfunction, reduced nitric oxide bioavailability, vascular stiffness, activation of the renin–angiotensin–aldosterone system, and accelerated atherosclerosis [31,45]. Consequently, visceral adiposity is strongly associated with hypertension, coronary artery disease, heart failure, and increased cardiovascular mortality. Visceral adiposity is a major driver of MetS through interconnected mechanisms involving insulin resistance, chronic inflammation, lipotoxicity, oxidative stress, and endocrine dysfunction [29,31,36].

### 3.4. Hormonal Imbalance

Hormonal imbalance represents a major pathophysiological component of MetS, reflecting the disruption of multiple endocrine pathways involved in energy homeostasis, glucose metabolism, adipose tissue regulation, appetite control, and inflammatory responses. MetS is not only a metabolic disorder but also a condition characterized by complex neuroendocrine dysfunction involving adipose tissue hormones, pancreatic hormones, HPA axis activity, sex hormones, and incretin signaling [21,36]. These hormonal disturbances interact closely with chronic low-grade inflammation, visceral obesity, insulin resistance, and gut microbiota alterations, thereby perpetuating metabolic dysfunction.

One of the major endocrine alterations in MetS involves adipokine dysregulation resulting from adipose tissue dysfunction. Reduced adiponectin levels impair insulin sensitivity and anti-inflammatory signaling, whereas elevated leptin concentrations are frequently accompanied by leptin resistance, leading to altered appetite regulation and promotion of systemic inflammation. Additional adipokines, including resistin, visfatin, and apelin, may further contribute to metabolic and vascular dysfunction. Collectively, these alterations link adipose tissue dysfunction with insulin resistance, chronic inflammation, and increased cardiometabolic risk [37,39,41,42,45,46].

Pancreatic hormonal imbalance is another central mechanism in MetS. In the early stages of insulin resistance, pancreatic β-cells compensate by increasing insulin secretion, leading to hyperinsulinemia [21]. Although initially adaptive, chronic hyperinsulinemia promotes lipogenesis, sodium retention, sympathetic nervous system activation, and further insulin resistance. Persistent metabolic stress eventually induces β-cell dysfunction through mechanisms involving glucotoxicity, lipotoxicity, mitochondrial dysfunction, oxidative stress, and inflammation [47,48]. As β-cell function progressively deteriorates, insulin secretion becomes insufficient, promoting the transition from MetS to type 2 diabetes mellitus.

In addition to insulin abnormalities, glucagon dysregulation contributes significantly to metabolic dysfunction. In healthy individuals, insulin suppresses glucagon secretion after meals. However, in insulin-resistant states, inappropriate glucagon secretion persists, increasing hepatic gluconeogenesis and hepatic glucose output [49]. Elevated glucagon levels therefore contribute to fasting hyperglycemia and impaired glucose homeostasis.

The HPA axis also plays a major role in hormonal imbalance associated with MetS. Chronic psychological stress, sleep disturbances, obesity, and inflammation may lead to persistent activation of the HPA axis and elevated cortisol secretion [50]. Cortisol exerts multiple metabolic effects that promote the development of MetS. It stimulates hepatic gluconeogenesis, increases proteolysis and lipolysis, promotes visceral fat accumulation, and impairs insulin sensitivity [51]. Increased local cortisol production within visceral adipose tissue is additionally mediated by 11β-hydroxysteroid dehydrogenase type 1 (11β-HSD1), an enzyme highly expressed in adipose tissue [52].

Chronic cortisol excess also contributes to appetite dysregulation and preference for calorie-dense foods rich in sugars and saturated fats [53]. Furthermore, cortisol interacts with inflammatory pathways and gut microbiota composition, reinforcing the bidirectional relationship between stress, endocrine dysfunction, and metabolic disease [54].

Alterations in sex hormone levels, including reduced testosterone in men and hyperandrogenism or estrogen deficiency in women, are associated with visceral adiposity, insulin resistance, and increased cardiometabolic risk [55,56,57,58]. Another important hormonal component involves incretin hormones, particularly glucagon-like peptide-1 (GLP-1) and glucose-dependent insulinotropic polypeptide (GIP), which regulate glucose homeostasis and appetite. Impaired incretin signaling contributes to defective insulin secretion and metabolic dysfunction, while emerging evidence suggests that gut microbiota dysbiosis may influence incretin release through alterations in SCFA production and enteroendocrine signaling [47,59,60].

In MetS and obesity, incretin signaling is frequently impaired. Reduced GLP-1 secretion and diminished incretin responsiveness contribute to defective insulin secretion, increased appetite, and impaired glucose regulation [59]. Emerging evidence also indicates that gut microbiota dysbiosis may influence incretin secretion through alterations in SCFA production and enteroendocrine signaling [60].

Hormonal imbalance in MetS is therefore highly interconnected with inflammatory, neural, and microbial pathways. Adipose tissue dysfunction, pancreatic stress, HPA axis activation, sex hormone alterations, and impaired incretin signaling collectively contribute to insulin resistance, visceral obesity, dyslipidemia, endothelial dysfunction, and chronic inflammation. These endocrine alterations perpetuate metabolic dysregulation and increase the risk of cardiovascular disease and type 2 diabetes mellitus. Hormonal dysregulation integrates metabolic, inflammatory, and neuroendocrine pathways, contributing to the development and progression of MetS.

**Figure 1:**
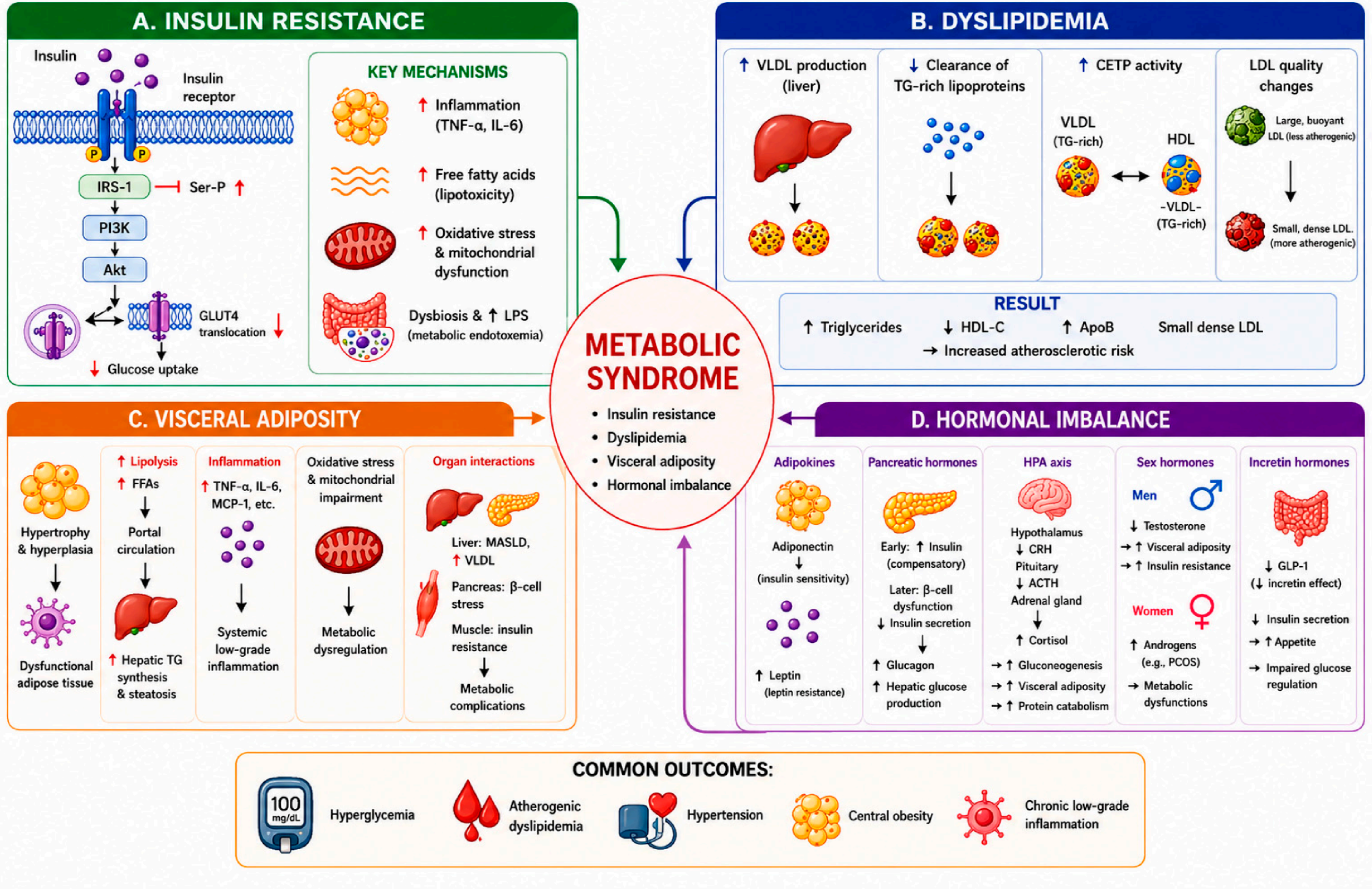
Overview of the major pathophysiological mechanisms underlying MetS. The figure summarizes the main interacting mechanisms involved in MetS development, including insulin resistance, visceral adiposity, dyslipidemia, chronic low-grade inflammation, hormonal imbalance, oxidative stress, mitochondrial dysfunction, and gut microbiota dysbiosis. These mechanisms reinforce each other and contribute to impaired glucose metabolism, lipid abnormalities, endothelial dysfunction, increased cardiovascular risk, and progression toward type 2 diabetes mellitus. For clarity, the figure presents the main mechanistic axes, whereas detailed molecular pathways and microbiota-related mechanisms are discussed in the text and summarized in the corresponding tables. Created in BioRender. Hangan, A.C. (2026) https://BioRender.com/6a5510563f5bbcdf74d59471, accessed on 10 June 2026.

Figure 1 provides a simplified overview of the major interconnected pathophysiological mechanisms involved in the development and progression of MetS.

### 3.5. Chronic Low-Grade Inflammation

Chronic low-grade inflammation is considered a central feature of MetS and represents one of the major mechanisms linking visceral obesity to insulin resistance, dyslipidemia, and cardiovascular disease. Unlike acute inflammation, which develops rapidly and resolves after the elimination of an injurious stimulus, low-grade inflammation is characterized by persistent activation of the immune system with mildly elevated circulating inflammatory mediators [43]. Although the inflammatory response is less intense than in infectious or autoimmune diseases, its chronic nature contributes significantly to long-term metabolic dysfunction.

The development of chronic inflammation in MetS is closely associated with excess visceral adipose tissue. Adipose tissue is no longer regarded as a passive energy storage site but rather as an active endocrine organ capable of producing hormones, cytokines, and adipokines that influence systemic metabolism [46]. Dysfunctional visceral adipose tissue is a major source of inflammatory mediators in MetS. As previously discussed, adipose tissue expansion is accompanied by increased cytokine production and immune activation, generating a persistent pro-inflammatory environment that contributes to systemic metabolic dysfunction [40,61]. Also, pro-inflammatory cytokines released from dysfunctional adipose tissue contribute to insulin resistance, altered lipid metabolism, and progressive metabolic dysfunction through activation of NF-κB and JNK signaling pathways [62,63].

Gut-derived inflammation further amplifies these processes through metabolic endotoxemia and activation of TLR4-dependent inflammatory pathways, as mentioned earlier [64]. The persistent inflammatory state observed in MetS also affects vascular function. Pro-inflammatory mediators impair endothelial homeostasis, reduce nitric oxide bioavailability, and promote endothelial dysfunction [65]. These changes contribute to hypertension, vascular stiffness, and accelerated atherosclerosis, which are major complications associated with MetS. Chronic low-grade inflammation links adipose tissue dysfunction, insulin resistance, and cardiovascular complications, representing a central mechanism in MetS pathogenesis.

**Figure 2:**
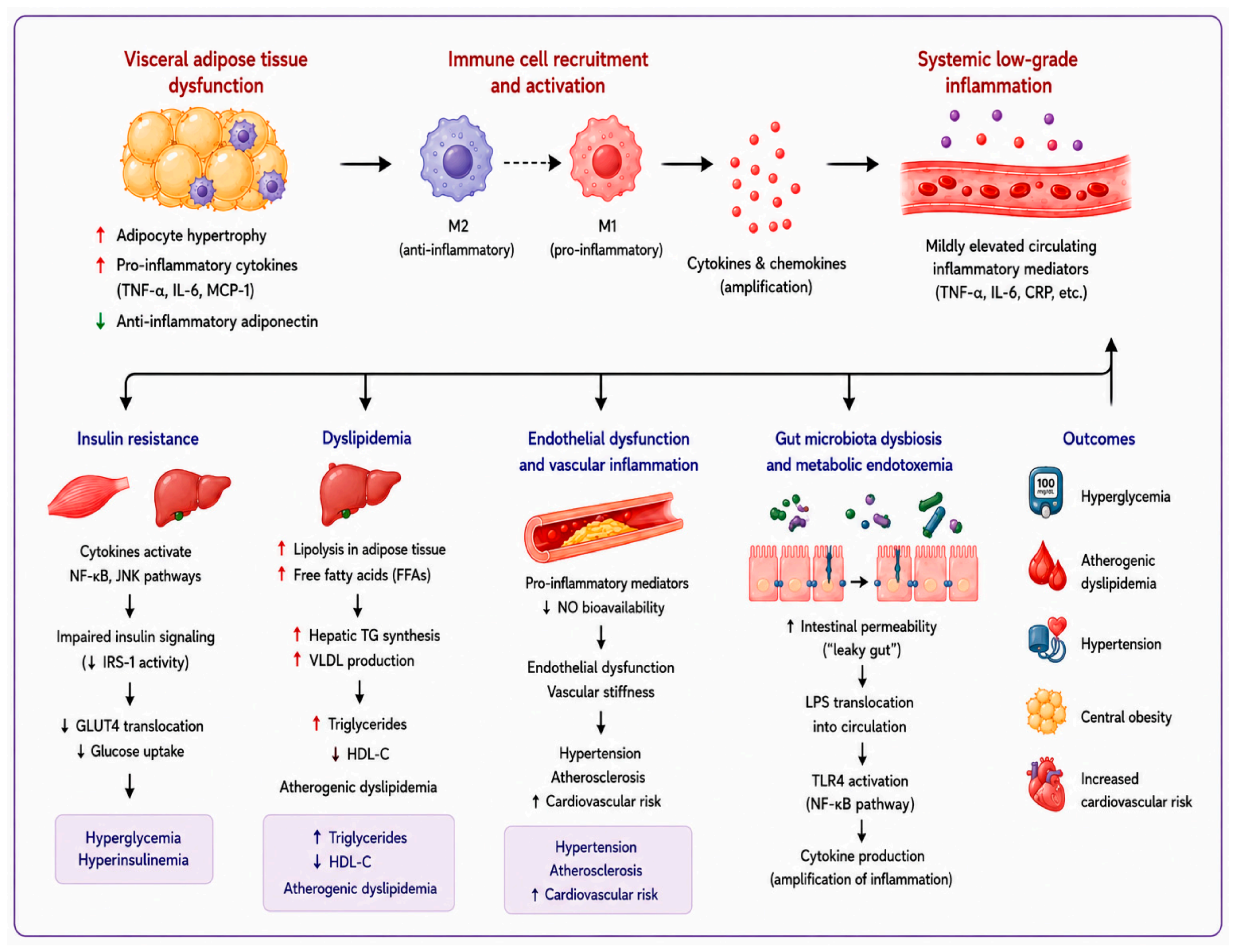
Representation of chronic low-grade inflammation as a central mechanism in MetS. Expansion of visceral adipose tissue promotes adipocyte hypertrophy, increased secretion of pro-inflammatory cytokines, macrophage recruitment, and reduced adiponectin production. These processes impair insulin signaling, lipid metabolism, endothelial function, and cardiovascular homeostasis. In parallel, gut microbiota dysbiosis and increased intestinal permeability facilitate LPS translocation and activation of TLR4/NF-κB-dependent inflammatory pathways, further sustaining systemic inflammation and metabolic dysfunction. For readability, the figure highlights the main inflammatory mechanisms, while detailed bacterial taxa, microbial metabolites, and biochemical pathways are presented in the text and tables. Created in BioRender. Hangan, A.C. (2026) https://BioRender.com/6a550fb5ac31fae9ec416bbf, accessed on 12 June 2026.

Figure 2 provides a simplified representation of the central role of chronic low-grade inflammation in MetS, emphasizing the interaction between visceral adipose tissue dysfunction, immune activation, gut microbiota dysbiosis, and cardiometabolic impairment.

## 4. Gut Microbiota, Gut–Brain Axis Signaling, and Gene–Microbiome Interactions in MetS

### 4.1. Gut Microbiota Dysbiosis and Metabolic Syndrome

#### 4.1.1. Gut Microbiota Composition, Microbial Metabolites, and Metabolic Homeostasis

The human gut microbiota is a highly complex and dynamic ecosystem composed of trillions of microorganisms whose collective genome significantly expands host metabolic capacity. At the phylum level, the microbiota is predominantly composed of *Firmicutes* and *Bacteroidetes*, which together account for more than 80–90% of the total bacterial population, while *Actinobacteria* (e.g., *Bifidobacterium*), *Proteobacteria*, and *Verrucomicrobia* (notably *Akkermansia muciniphila*) are present in smaller but functionally important proportions [66,67,68]. The relative abundance of these groups is highly variable and influenced by diet, age, antibiotic exposure, and host genetics. Recent evidence also highlights that microbiota composition is highly dynamic and can rapidly respond to dietary interventions, with functional changes often preceding taxonomic shifts [69].

A major function of the gut microbiota is the fermentation of non-digestible dietary substrates into SCFAs, primarily acetate, propionate, and butyrate. These metabolites contribute to intestinal barrier integrity, immune regulation, and metabolic homeostasis, while also participating in gut–brain communication. The microbiota also metabolizes dietary proteins, generating bioactive compounds that may influence intestinal and systemic physiology [70,71,72].

Beyond digestion, the gut microbiota functions as a central regulator of host metabolism. SCFAs act as signaling molecules through G-protein-coupled receptors such as GPR41, GPR43, and GPR109A, influencing insulin sensitivity, adipocyte differentiation, and the secretion of enteroendocrine hormones, including GLP-1 and peptide YY (PYY), which regulate appetite and glucose homeostasis [73]. The microbiota also modulates bile acid metabolism by converting primary bile acids into secondary bile acids, thereby regulating signaling pathways mediated by nuclear receptors such as the farnesoid X receptor (FXR) and G protein-coupled bile acid receptor 1 (GBPAR1). These signaling cascades play critical roles in lipid metabolism, glucose regulation, and energy expenditure [74,75]. Furthermore, microbial metabolites such as trimethylamine (TMA), derived from dietary choline and carnitine, are converted in the liver to trimethylamine N-oxide (TMAO), which has been strongly linked in recent studies to atherosclerosis, cardiovascular disease, and MetS progression [76,77].

The gut microbiota is also essential for immune system development and homeostasis. It shapes both innate and adaptive immunity through continuous interaction with the mucosal immune system. Commensal bacteria stimulate the maturation of gut-associated lymphoid tissue and promote the differentiation of regulatory T cells (Tregs), which are crucial for maintaining immune tolerance and preventing excessive inflammation. Microbial-associated molecular patterns interact with pattern recognition receptors such as toll-like receptors and NOD-like receptors (NLRs), ensuring a balanced immune response [78,79]. Certain commensal species, such as *Faecalibacterium prausnitzii*, exert anti-inflammatory effects through the production of butyrate and other metabolites, whereas an increased abundance of *Proteobacteria* is often associated with pro-inflammatory states and metabolic disturbances [66,67].

The integrity of the intestinal barrier is tightly regulated by the microbiota. Beneficial microorganisms enhance mucus layer thickness, stimulate antimicrobial peptide production, and strengthen epithelial tight junctions, thereby preventing pathogen translocation. *Akkermansia muciniphila*, in particular, plays a key role in maintaining mucosal integrity and has been inversely associated with obesity, insulin resistance, and MetS [29,80]. In contrast, dysbiosis can disrupt barrier function, leading to increased intestinal permeability (“leaky gut”). This allows bacterial components such as LPS to enter the systemic circulation, a phenomenon known as metabolic endotoxemia. LPS activates inflammatory pathways via TLR4 signaling, contributing to chronic low-grade inflammation, insulin resistance, and metabolic dysfunction [64]. The gut microbiota plays a central role in metabolic and immune homeostasis, and alterations in its composition and function may contribute to the development of MetS through multiple interconnected mechanisms [73,77,80].

To provide a more specific mechanistic overview, representative bacterial taxa, microbial metabolites, and biochemical pathways involved in gut–brain–metabolic signaling are summarized in Table 1. These examples illustrate how dysbiosis may contribute to MetS not only through broad changes in microbial diversity but also through specific microbial products, including SCFAs, LPS, bile acid derivatives, TMAO, tryptophan metabolites, and neurotransmitter-related compounds. These metabolites interact with host receptors and signaling pathways, including FFAR2/3, GPR109A, FXR, TGR5, TLR4/NF-κB, MAPK, AhR, enteroendocrine hormone secretion, vagal signaling, and HPA axis regulation.

These taxa and metabolites indicate that the contribution of the gut microbiota to MetS is mediated by specific biochemical pathways rather than by nonspecific dysbiosis alone. In particular, SCFA signaling, LPS/TLR4-mediated inflammation, bile acid receptor signaling, TMAO production, tryptophan metabolism, and microbiota-related epigenetic regulation provide mechanistic links between gut microbial alterations, gut–brain axis dysregulation, and metabolic impairment.

#### 4.1.2. Lifestyle, Dietary, and Environmental Determinants of Gut Microbiota Composition and Function

The composition and functional capacity of the gut microbiota are continuously shaped by a complex interplay of environmental exposures and host-related factors, among which diet, antibiotic use, and lifestyle behaviors represent the most influential and modifiable determinants. These factors affect not only microbial diversity and taxonomic composition but also the metabolic output of the microbiota, thereby influencing host physiology, immune responses, and susceptibility to metabolic diseases.

Diet is the dominant factor governing microbiota structure and function, exerting both short-term and long-term effects. Acute dietary changes can rapidly alter microbial gene expression and metabolite production within 24–48 h, even before significant taxonomic shifts occur, highlighting the functional plasticity of the microbiome [66]. Long-term dietary patterns, however, determine stable microbial configurations. Diets rich in plant-based foods, dietary fiber, and polyphenols promote the expansion of beneficial taxa such as *Bifidobacterium*, *Lactobacillus*, and butyrate-producing bacteria, including *Faecalibacterium prausnitzii* and *Roseburia* spp., which enhance SCFA production and support epithelial integrity, immune tolerance, and metabolic regulation [67,71]. Polyphenols, in particular, undergo microbial biotransformation into bioactive metabolites that exert antioxidant and anti-inflammatory effects, further reinforcing host–microbiota symbiosis [99].

In contrast, Western-style diets characterized by high intake of saturated fats, refined carbohydrates, and low fiber are consistently associated with reduced microbial diversity, depletion of beneficial SCFA-producing bacteria, and expansion of pro-inflammatory taxa such as *Proteobacteria* [66,69]. High-fat diets also alter bile acid composition, increasing the proportion of secondary bile acids that favor the growth of bile-tolerant microorganisms such as *Bilophila wadsworthia*, which has been linked to intestinal inflammation and metabolic dysfunction [88]. Moreover, diets rich in red meat and animal-derived nutrients increase microbial production of TMA, which is subsequently converted into TMAO in the liver. More specifically, TMAO production represents a diet–microbiome–liver pathway. Dietary choline, phosphatidylcholine, and L-carnitine, mainly derived from red meat, eggs, dairy products, and other animal-based foods, act as TMAO-related precursors. These substrates are metabolized by specific gut bacterial enzymes into trimethylamine (TMA), which is absorbed through the intestinal epithelium and transported via the portal circulation to the liver. In hepatocytes, TMA is oxidized by flavin-containing monooxygenases, particularly FMO3, to form trimethylamine N-oxide (TMAO). Increased circulating TMAO may contribute to MetS progression by promoting endothelial dysfunction, vascular inflammation, altered cholesterol and bile acid metabolism, platelet activation, atherosclerosis, insulin resistance, and cardiovascular risk [76,77,87,100].

Recent studies also emphasize that dietary patterns influence circadian oscillations of the microbiome, affecting diurnal fluctuations in microbial metabolites and host metabolic pathways, which may contribute to metabolic disorders when disrupted [101].

Antibiotic exposure is another major determinant of microbiota composition, often leading to profound and sometimes long-lasting alterations. Antibiotics reduce microbial diversity, disrupt ecological balance, and can eliminate keystone species critical for maintaining metabolic and immune homeostasis. Although partial recovery may occur after antibiotic cessation, the microbiota often fails to fully return to its original state, particularly after repeated exposures [102]. Early-life antibiotic use is especially impactful, as it interferes with the establishment of a stable and diverse microbiome during critical developmental windows. This disruption has been associated with increased long-term risk of obesity, asthma, allergies, and MetS [103]. Mechanistically, antibiotic-induced dysbiosis leads to reduced SCFA production, impaired bile acid metabolism, and disruption of intestinal barrier integrity, thereby facilitating systemic inflammation and metabolic dysfunction [104].

Lifestyle factors further modulate the microbiome through multiple interconnected pathways. Regular physical activity has been shown to increase microbial diversity and enrich SCFA-producing bacteria, contributing to improved metabolic and inflammatory profiles. Exercise-induced changes in the microbiome are associated with enhanced mitochondrial function, improved insulin sensitivity, and increased production of beneficial metabolites such as butyrate [105,106]. In contrast, sedentary behavior is associated with reduced microbial diversity and a metabolic profile linked to increased disease risk.

Sleep patterns and circadian rhythms also play a critical role in shaping the microbiome. Disruptions such as sleep deprivation or shift work alter microbial composition and reduce rhythmicity in microbial metabolite production, which can impair glucose metabolism and promote inflammation [101,107]. These effects are mediated through interactions between the microbiome and host circadian genes, illustrating a bidirectional relationship between microbial and host metabolic cycles. Psychological stress influences the microbiota through neuroendocrine mechanisms involving activation of the HPA axis, alterations in gut permeability, and changes in microbial composition, thereby promoting dysbiosis and low-grade inflammation [108].

Smoking has been associated with reduced microbial diversity, enrichment of pro-inflammatory bacterial taxa, and increased systemic inflammation, potentially contributing to metabolic dysfunction and cardiometabolic risk [109]. Excessive alcohol consumption disrupts gut microbiota composition, impairs intestinal barrier integrity, and promotes endotoxemia, thereby contributing to metabolic dysfunction [110].

Collectively, diet, antibiotic exposure, physical activity, sleep quality, and psychological stress are major determinants of microbiota composition and function. Through their effects on microbial diversity and metabolite production, these factors influence metabolic homeostasis and susceptibility to MetS [66,67,103,105].

Table 2 summarizes selected observational, evidence-synthesis, and interventional studies addressing lifestyle-related factors, gut microbiota alterations, and MetS-related outcomes. The table was reorganized to distinguish lifestyle exposures, study type, microbiota-related findings, metabolic relevance, and methodological limitations.

#### 4.1.3. Microbial Metabolites, Barrier Dysfunction, and Inflammatory Pathways Linking Dysbiosis to MetS

As highlighted in the previous sections, gut microbiota dysbiosis has emerged as an important contributor to the development and progression of MetS, particularly through its involvement in intestinal permeability alterations, systemic inflammation, and metabolic dysregulation.

Dysbiosis refers to qualitative and quantitative alterations in the composition, diversity, and metabolic activity of the intestinal microbiota that disrupt the physiological balance between the host and gut microorganisms [115]. Under normal conditions, the gut microbiota contributes to nutrient metabolism, immune regulation, maintenance of intestinal barrier integrity, and production of metabolites involved in metabolic homeostasis. However, disturbances in microbial composition may promote obesity, insulin resistance, dyslipidemia, and chronic inflammation.

In healthy individuals, the intestinal microbiota is dominated mainly by bacteria belonging to the *Firmicutes* and *Bacteroidetes phyla*, together with smaller proportions of *Actinobacteria* and *Proteobacteria* [66]. Dysbiosis is frequently associated with reduced microbial diversity and alterations in the relative abundance of these bacterial groups. Western dietary patterns rich in saturated fats, refined carbohydrates, and ultra-processed foods have been associated with decreased levels of beneficial bacteria such as *Bifidobacterium* and *Faecalibacterium prausnitzii*, both of which are involved in anti-inflammatory activity and SCFA production [83].

One of the most important mechanisms linking dysbiosis to MetS is impairment of intestinal barrier integrity. Under physiological conditions, intestinal epithelial cells are connected by tight junction proteins that regulate intestinal permeability and prevent harmful bacterial products from entering systemic circulation [116]. Dysbiosis may disrupt the expression and function of tight junction proteins, resulting in increased intestinal permeability, commonly referred to as “leaky gut.”

As intestinal permeability increases, bacterial LPS, particularly those derived from Gram-negative bacteria, may translocate into the bloodstream [64]. This process, known as metabolic endotoxemia, contributes significantly to systemic inflammation associated with MetS. Once in circulation, LPS activates TLR4-mediated inflammatory pathways in immune cells, adipose tissue, skeletal muscle, and the liver [89]. Activation of these pathways stimulates nuclear NF-κB, promoting the release of pro-inflammatory cytokines such as TNF-α, IL-6, and IL-1β.

The persistent production of inflammatory mediators contributes to chronic low-grade systemic inflammation, which represents a central feature of MetS [90]. Pro-inflammatory cytokines interfere with insulin signaling pathways by impairing IRS-1 activity and reducing insulin-mediated glucose uptake in peripheral tissues. Consequently, insulin resistance develops in skeletal muscle, adipose tissue, and the liver, promoting hyperglycemia and compensatory hyperinsulinemia. In addition to inflammatory activation, dysbiosis also affects the production of microbial metabolites, particularly SCFAs. SCFAs, mainly acetate, propionate, and butyrate, are generated through bacterial fermentation of dietary fiber [81]. These metabolites exert multiple beneficial effects on host metabolism. They contribute to maintenance of intestinal barrier integrity, modulation of immune responses, regulation of glucose and lipid metabolism, and control of appetite and satiety.

Among SCFAs, butyrate plays a particularly important role because it serves as the main energy source for colonocytes and supports tight junction stability [81]. Butyrate also exhibits anti-inflammatory properties by reducing pro-inflammatory cytokine production and promoting regulatory immune responses. Reduced abundance of butyrate-producing bacteria in dysbiosis may therefore contribute to increased intestinal permeability, inflammation, and impaired insulin sensitivity. SCFAs additionally influence metabolic regulation through interaction with the GBA. They stimulate the secretion of gut hormones such as GLP-1 and PYY, which regulate appetite, satiety, and glucose homeostasis [117]. Altered SCFA production in dysbiosis may therefore contribute to excessive caloric intake, weight gain, and visceral obesity. The effects of SCFAs can be organized into several interconnected mechanistic cascades. First, through intestinal barrier-related pathways, especially butyrate supports colonocyte energy metabolism, tight junction integrity, mucus production, and epithelial defense, thereby reducing intestinal permeability and limiting LPS translocation. Second, through immune-inflammatory pathways, SCFAs may promote regulatory T-cell differentiation, inhibit excessive NF-κB activation, and reduce pro-inflammatory cytokine production. Third, through enteroendocrine and metabolic pathways, acetate, propionate, and butyrate activate FFAR2/3 and GPR109A signaling, stimulate GLP-1 and PYY secretion, improve insulin sensitivity, and contribute to appetite and glucose regulation. Fourth, through gut–brain axis-related pathways, SCFAs may influence vagal signaling, neuroendocrine responses, and appetite-regulating circuits [73,81,82,117,118].

Dysbiosis may also affect bile acid metabolism and energy extraction from the diet. Certain microbial profiles increase the efficiency of energy harvest from indigestible carbohydrates, thereby promoting adiposity and fat accumulation [104]. Altered bile acid metabolism may further impair lipid and glucose homeostasis, contributing to dyslipidemia and insulin resistance. At the biochemical level, these effects are mediated by changes in microbial products rather than by taxonomic imbalance alone. Reduced production of butyrate and other SCFAs may impair epithelial barrier integrity, GLP-1/PYY secretion, and anti-inflammatory signaling, whereas increased LPS availability from Gram-negative bacteria activates TLR4/NF-κB-dependent inflammation. In parallel, altered bile acid transformation, increased TMAO generation, and disrupted tryptophan metabolism may affect lipid metabolism, endothelial function, appetite regulation, HPA axis activity, and gut–brain communication.

Collectively, these findings support a central role for dysbiosis in the development of MetS through its effects on intestinal permeability, inflammation, and metabolic regulation.

#### 4.1.4. Limitations of the Current Evidence

Although current evidence supports a relevant contribution of the gut microbiota and the gut–brain axis to metabolic syndrome, the strength of this evidence varies considerably across study types. Much of the mechanistic knowledge comes from preclinical and animal models, which are useful for identifying biological pathways but have important translational limitations. Differences between experimental models and human metabolic disease, including microbiota composition, dietary exposure, host genetic background, environmental influences, and study design, may explain why some findings are not consistently reproduced in clinical settings.

In human studies, the evidence is still largely based on observational and cross-sectional data. Therefore, associations between dysbiosis, microbial metabolites, inflammation, and metabolic syndrome should be interpreted with caution, as they do not necessarily demonstrate causality. These associations may be affected by several confounding factors, including diet, medication use, age, degree of obesity, physical activity, and associated comorbidities. In addition, heterogeneity in sequencing techniques, bioinformatic analysis, sample collection protocols, and population characteristics may contribute to differences between studies and limit reproducibility.

Clinical trials investigating microbiota-targeted interventions, such as probiotics, prebiotics, dietary modulation, or fecal microbiota transplantation, are still relatively limited and heterogeneous. Their results may be influenced by baseline microbiota composition, intervention duration, strain specificity, adherence, dietary background, and metabolic phenotype. Thus, while microbiota-based strategies remain promising, their routine clinical application in metabolic syndrome requires further well-designed, adequately powered, and independently reproducible human studies.

### 4.2. Gut–Brain Axis Mechanisms and Metabolic Dysregulation in MetS

The gut–brain axis represents a bidirectional communication network linking the gastrointestinal tract, gut microbiota, enteric nervous system, vagus nerve, immune system, endocrine signaling, and central nervous system. In MetS, this axis is not disrupted through a single pathway but through coordinated alterations in neural, endocrine, immune, metabolic, and microbial signaling. The vagus nerve provides the most direct and rapid communication route, with most vagal fibers transmitting afferent signals from the gut to the brain, while efferent fibers regulate intestinal motility, secretion, barrier function, and immune responses [103]. Vagal afferents respond indirectly to microbial and luminal signals through host mediators such as SCFAs, bile acid derivatives, serotonin, GABA, cytokines, and peptide hormones, which are transmitted to brain regions involved in appetite, satiety, mood, autonomic regulation, and energy balance [81,119,120]. In this context, SCFAs influence GBA signaling not only as microbial metabolites but also through barrier-protective, immune-inflammatory, enteroendocrine, and vagal pathways [121].

At the biochemical level, this communication is mediated by specific microbial products rather than by dysbiosis in general. SCFAs produced by butyrate-generating bacteria such as *Faecalibacterium prausnitzii*, *Roseburia* spp., and *Eubacterium rectale* may stimulate FFAR2/3-dependent enteroendocrine signaling and GLP-1/PYY secretion, whereas LPS derived mainly from Gram-negative bacteria activates TLR4/NF-κB-dependent inflammatory pathways. In parallel, microbial bile acid derivatives, TMAO, indole derivatives, and neurotransmitter-related metabolites may influence FXR/TGR5 signaling, endothelial function, immune activation, vagal signaling, HPA axis activity, appetite regulation, and metabolic homeostasis.

Recent evidence also shows that microbiota composition can influence vagal tone, whereas vagal modulation may affect microbial composition, metabolite production, and central signaling pathways involved in neuroplasticity and metabolic regulation [122,123].

Enteroendocrine cells represent a central interface between microbial, endocrine, and neural signaling. Although they account for a small proportion of the intestinal epithelium, they detect nutrients, SCFAs, bile acids, and other microbial metabolites through receptors such as FFAR2/3 and TGR5, leading to secretion of hormones including GLP-1, PYY, CCK, serotonin, and ghrelin [60,124,125,126,127]. These hormones regulate appetite, satiety, glucose metabolism, intestinal motility, and energy balance. Some enteroendocrine subsets, known as neuropod cells, form synapse-like contacts with vagal afferents and release neurotransmitters such as glutamate, allowing rapid gut-to-brain communication [128]. Thus, enteroendocrine signaling should be viewed as a hybrid neuroendocrine mechanism that connects microbial metabolites with vagal activation, hormonal responses, and metabolic regulation. In addition, microbial metabolites may influence host gene expression through epigenetic mechanisms, including histone modification and DNA methylation, thereby linking microbiota activity to longer-term metabolic and neuroendocrine regulation [129].

Stress-related neuroendocrine signaling provides another important component of GBA dysregulation in MetS. Activation of the HPA axis leads to cortisol secretion, which can influence intestinal motility, mucus secretion, epithelial permeability, immune responses, and gut microbiota composition [54,60,130,131]. Conversely, dysbiosis and increased intestinal permeability may promote LPS translocation and cytokine production, including IL-1β, IL-6, and TNF-α, which can further stimulate hypothalamic CRH release and HPA axis activation [54,60,130]. This bidirectional interaction links psychological stress, microbial imbalance, gut barrier dysfunction, and metabolic inflammation.

Immune signaling connects dysbiosis with systemic metabolic inflammation. Under physiological conditions, the intestinal immune system maintains tolerance toward commensal microorganisms through epithelial barrier integrity, secretory IgA, regulatory T cells, and anti-inflammatory cytokines [6,132,133]. Dysbiosis may reduce SCFA production and weaken tight junction proteins, increasing intestinal permeability and allowing bacterial products such as LPS, peptidoglycans, flagellin, and bacterial DNA to cross the epithelial barrier [64,116,134]. These microbial products activate pattern-recognition receptors, particularly TLR4, and downstream NF-κB and MAPK signaling, leading to increased production of TNF-α, IL-1β, IL-6, IL-17, and IFN-γ [64,135]. This chronic low-grade inflammatory state contributes to insulin resistance, adipose tissue dysfunction, endothelial dysfunction, and metabolic impairment. The NLRP3 inflammasome further links microbial, metabolic, and inflammatory signals. LPS, oxidative stress, mitochondrial dysfunction, saturated fatty acids, ceramides, and other danger signals can activate NLRP3 in immune cells, promoting maturation of IL-1β and IL-18 and amplifying systemic inflammation [134,135,136,137,138]. In contrast, SCFAs, particularly butyrate, may limit inflammation by strengthening epithelial barrier function, inhibiting NF-κB signaling, promoting regulatory T-cell differentiation, and reducing inflammasome activation [132,135]. The immune pathway also interacts with neural and endocrine mechanisms, since cytokines can activate the HPA axis, while vagal efferent signaling may suppress cytokine production through the cholinergic anti-inflammatory reflex [135,139].

Gut-derived neurotransmitters provide an additional biochemical link between microbiota activity, intestinal function, immunity, and brain-related metabolic regulation. Serotonin is the best-characterized example, with most peripheral serotonin produced in the gastrointestinal tract by enterochromaffin cells [84,85]. Microbial metabolites, including SCFAs and secondary bile acids, may stimulate serotonin synthesis and release, while dysbiosis and inflammation can shift tryptophan metabolism toward the kynurenine pathway, reducing serotonin availability and generating metabolites involved in inflammation, oxidative stress, and altered neurotransmission [84,85,86,91,92,93,94,140]. Other microbial or microbiota-modulated neurotransmitters, including GABA, glutamate, dopamine-related compounds, acetylcholine, and catecholamine-like metabolites, may act locally on enteric neurons, immune cells, epithelial cells, and vagal afferents [84,86,125,127]. In MetS, dysbiosis, chronic low-grade inflammation, altered microbial metabolite production, and impaired barrier integrity may disrupt gut-derived neurotransmitter signaling. These alterations can influence appetite regulation, intestinal transit, glucose homeostasis, immune activation, stress-related neuroendocrine responses, and gut–brain communication. Therefore, gut-derived neurotransmitters should be interpreted as part of the broader integrated GBA network rather than as isolated mediators.

Taken together, GBA dysregulation in MetS reflects the coordinated disruption of neural, endocrine, immune, metabolic, and microbial signaling. Gut dysbiosis alters microbial metabolite production, weakens intestinal barrier integrity, promotes systemic inflammation, modifies gut hormone and neurotransmitter signaling, and affects vagal and HPA axis activity. These interconnected pathways contribute to appetite dysregulation, insulin resistance, chronic low-grade inflammation, visceral adiposity, and metabolic dysfunction, supporting the role of the GBA as an integrated mechanism in MetS pathogenesis.

**Figure 3:**
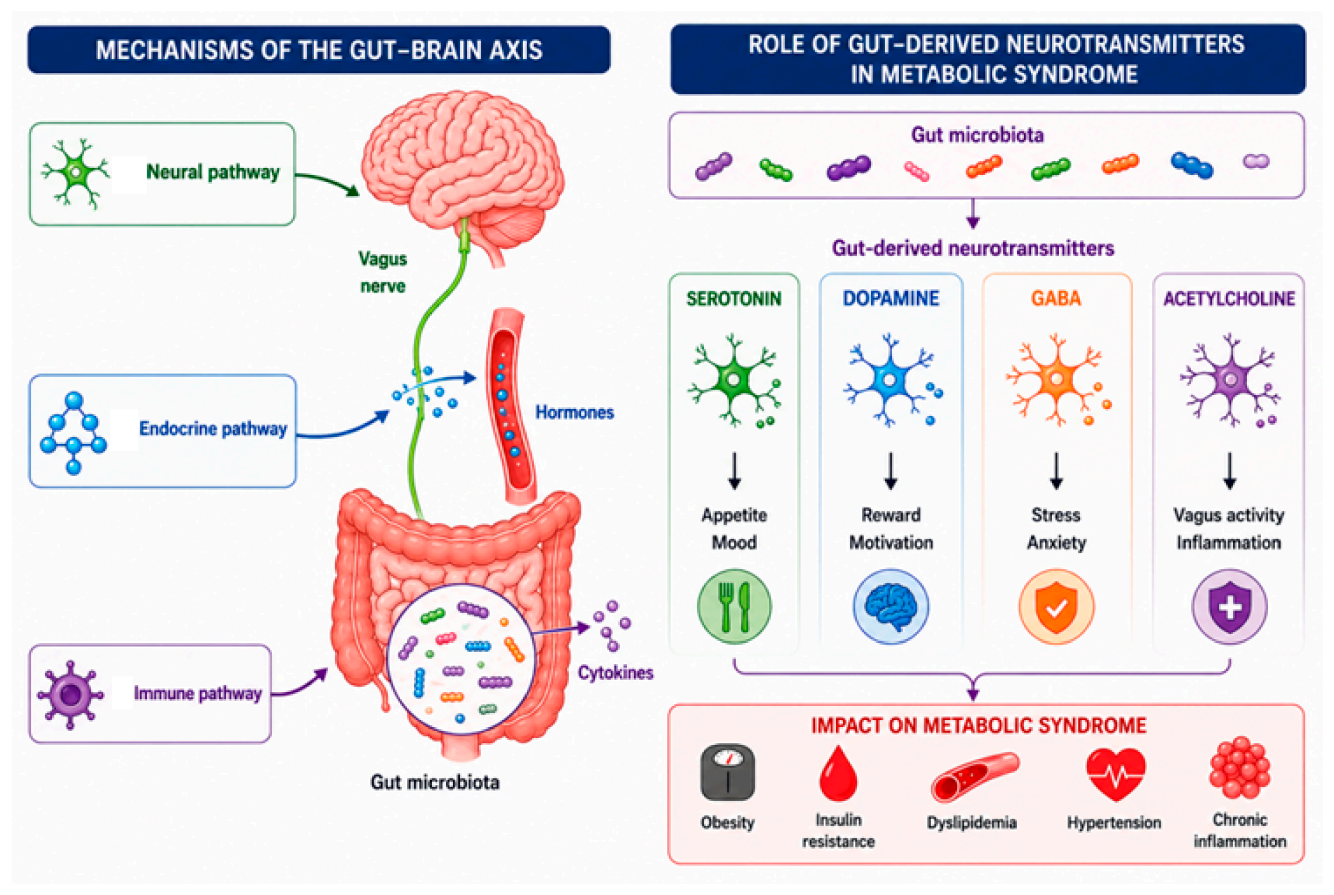
Overview of gut–brain axis signaling pathways involved in MetS. The figure summarizes the main bidirectional communication routes between the gut microbiota and the central nervous system, including neural, endocrine, immune, and neurotransmitter-related pathways. Specific microbial taxa and metabolites involved in these interactions include SCFA-producing bacteria, *Akkermansia muciniphila, Proteobacteria/Enterobacteriaceae*-derived LPS, bile acid-transforming bacteria, TMA-producing taxa, tryptophan-metabolizing bacteria, SCFAs, LPS, bile acid derivatives, TMAO, indole derivatives, serotonin, dopamine, GABA, and acetylcholine. These pathways may influence appetite regulation, stress responses, vagal activity, enteroendocrine hormone secretion, inflammation, insulin resistance, dyslipidemia, hypertension, and chronic low-grade inflammation. For clarity, the figure presents the main GBA signaling routes, whereas detailed bacterial taxa, metabolites, and receptor-mediated pathways are summarized in the text and tables. Created in BioRender. Hangan, A.C. (2026) https://BioRender.com/6a550c60720f248e415e6cec, accessed on 15 June 2026.

Figure 3 provides a simplified overview of the main GBA signaling routes involved in MetS, focusing on neural, endocrine, immune, and neurotransmitter-related communication between the gut microbiota and the central nervous system.

### 4.3. Genetic and Epigenetic Interactions

#### 4.3.1. Genetic Predisposition in Metabolic Syndrome

Genetic predisposition plays an important role in the development of MetS, particularly through genes involved in appetite regulation, adipose tissue distribution, insulin signaling, glucose metabolism, inflammation, and lipid homeostasis. Although environmental factors such as diet, sedentary lifestyle, smoking, and alcohol consumption are major contributors to MetS, genetic susceptibility may increase the vulnerability of certain individuals to obesity, insulin resistance, dyslipidemia, and type 2 diabetes mellitus [141]. MetS is therefore considered a multifactorial disorder resulting from the interaction between genetic and environmental determinants.

One of the most extensively studied obesity-related genes is the fat mass and obesity-associated gene (FTO). The FTO gene encodes an enzyme involved in nucleic acid demethylation and energy homeostasis regulation. It is highly expressed in the hypothalamus, a brain region involved in appetite control and food intake regulation [142]. The most studied polymorphism of this gene is rs9939609. Individuals carrying the A risk allele have been shown to present increased BMI, higher caloric intake, greater appetite, and increased risk of central obesity [142,143]. The mechanisms proposed include altered satiety signaling and preference for energy-dense foods. Since abdominal obesity is a core component of MetS, FTO polymorphisms indirectly contribute to MetS development through increased adiposity and subsequent insulin resistance. Adipose tissue accumulation, particularly visceral fat, promotes chronic low-grade inflammation and increased secretion of pro-inflammatory cytokines such as TNF-α and IL-6. These inflammatory mediators interfere with insulin receptor signaling pathways, thereby contributing to insulin resistance [141].

The transcription factor 7-like 2 (*TCF7L2*) gene is strongly associated with glucose metabolism and type 2 diabetes susceptibility. *TCF7L2* encodes a transcription factor involved in the Wnt signaling pathway, which regulates pancreatic β-cell function, insulin secretion, and glucose homeostasis. Among its polymorphisms, rs7903146 is the most studied variant associated with impaired glucose tolerance and diabetes risk. Carriers of the T-risk allele exhibit reduced insulin secretion and impaired β-cell function [144,145]. This polymorphism may also influence hepatic glucose production and insulin sensitivity. In MetS, impaired insulin secretion combined with peripheral insulin resistance contributes to chronic hyperglycemia and altered lipid metabolism. Studies have also suggested that *TCF7L2* variants may affect the response to dietary interventions, explaining part of the interindividual variability observed in metabolic outcomes [145].

The peroxisome proliferator-activated receptor gamma (*PPARG*) gene encodes a nuclear receptor that plays a major role in adipocyte differentiation, lipid storage, glucose metabolism, and insulin sensitivity [146]. *PPARG* regulates the expression of genes involved in fatty acid uptake and adipogenesis and is highly expressed in adipose tissue. The *Pro12Ala* polymorphism (rs1801282) is one of the most frequently investigated variants of *PPARG*. Some studies suggest that the Ala allele may be associated with improved insulin sensitivity and lower risk of type 2 diabetes, whereas other reports indicate that the metabolic effects of this polymorphism may depend on environmental factors such as dietary fat intake and obesity status [141,146]. Dysregulation of PPARG activity may lead to abnormal adipocyte function, ectopic fat accumulation, and altered secretion of adipokines, all of which contribute to insulin resistance and metabolic dysfunction.

The adiponectin gene (ADIPOQ) encodes adiponectin, an adipokine secreted predominantly by adipose tissue. Adiponectin exerts anti-inflammatory, anti-atherogenic, and insulin-sensitizing effects. It enhances fatty acid oxidation, improves glucose uptake in skeletal muscle, and suppresses hepatic glucose production. Lower adiponectin levels are commonly observed in obesity and MetS. Polymorphisms such as rs2241766 and rs1501299 have been associated with reduced adiponectin concentrations, increased insulin resistance, dyslipidemia, and higher susceptibility to MetS [147]. Reduced adiponectin activity contributes to chronic inflammation and impaired insulin signaling, thereby promoting the development of MetS components.

The APOA5 gene is involved in triglyceride metabolism. APOA5 protein regulates plasma triglyceride concentrations by influencing lipoprotein lipase activity and VLDL metabolism. The rs662799 polymorphism has been associated with elevated triglyceride levels and increased risk of MetS [148,149]. Hypertriglyceridemia contributes to lipid accumulation in tissues such as the liver and skeletal muscle, which may impair insulin signaling pathways and exacerbate insulin resistance. Because dyslipidemia is one of the diagnostic criteria of MetS, APOA5 variants are considered important genetic contributors to metabolic dysfunction.

The melanocortin-4 receptor (*MC4R*) gene plays an important role in appetite regulation and energy expenditure. *MC4R* encodes a receptor expressed mainly in the hypothalamus, where it participates in the leptin–melanocortin signaling pathway controlling hunger and satiety. The rs17782313 polymorphism located near the *MC4R* gene has been associated with increased appetite, obesity risk, hyperglycemia, and insulin resistance [150]. Alterations in *MC4R* signaling may promote excessive food intake and reduced energy expenditure, thereby contributing to weight gain and metabolic disturbances.

Genetic polymorphisms may partly explain why individuals exposed to similar environmental conditions develop different metabolic outcomes. For example, some individuals carrying FTO or MC4R risk alleles may be more susceptible to obesity despite similar caloric intake, whereas variants in *TCF7L2* may predispose individuals to impaired insulin secretion and diabetes development [143,145,150].

Interindividual variability is also relevant in the context of lifestyle interventions. Nutrigenetic studies suggest that the response to dietary modifications, physical activity, or weight-loss programs may vary according to genotype. For instance, carriers of *TCF7L2* risk alleles may show different glycemic responses to carbohydrate intake, while *APOA5* polymorphisms may influence triglyceride responses to dietary fat reduction [145,149].

Therefore, genetic predisposition does not independently determine the occurrence of MetS but rather modulates susceptibility and metabolic response to environmental factors. Understanding these mechanisms may support the development of personalized prevention and treatment strategies targeting both lifestyle and genetic background.

#### 4.3.2. Host Genetics, Epigenetic Regulation, and Gene–Microbiome Interactions in MetS

Host genetics, epigenetic regulation, and gut microbiota should be considered interconnected components of the same regulatory network in MetS rather than independent mechanisms [97,151,152]. Host genetic variants may influence microbiota composition through effects on immune recognition, intestinal barrier integrity, mucosal glycosylation, lipid metabolism, appetite regulation, and inflammatory signaling [153,154,155,156]. Conversely, gut microorganisms and microbiota-derived metabolites may modulate host metabolic and immune pathways and influence gene expression through epigenetic mechanisms, including DNA methylation, histone modifications, and regulation of non-coding RNAs [95,96,151,152].

The composition and metabolic activity of the gut microbiota are influenced by both environmental and host-related factors, including diet, antibiotic exposure, lifestyle, age, immune status, and genetic background [155]. Host genetic factors may shape microbial diversity and the abundance of specific bacterial taxa by influencing intestinal barrier integrity, immune signaling, mucus secretion, nutrient availability, antimicrobial peptide production, and inflammatory responses. One of the major mechanisms through which host genes influence the microbiome involves the regulation of immune responses and intestinal barrier function. Genes involved in microbial recognition and innate immunity, such as NOD2, TLR4, and FUT2, contribute to the selection and maintenance of specific microbial populations [157]. Variants in these genes may alter microbial diversity, weaken host–microbial tolerance, and predispose individuals to dysbiosis and inflammation. In MetS, these mechanisms are particularly relevant because they may partly explain why individuals exposed to similar dietary or lifestyle conditions develop different metabolic phenotypes [66].

Host DNA influences gut microbiota composition through several interconnected physiological mechanisms. One of the most important mechanisms involves immune system regulation. The intestinal immune system constantly interacts with trillions of microorganisms and must maintain a balance between tolerance toward commensal bacteria and defense against pathogens [156]. Genes involved in innate immunity and microbial recognition are therefore essential determinants of microbial colonization patterns.

Among the most studied genes involved in host–microbiota interactions is the Fucosyltransferase 2 (*FUT2*) gene. *FUT2* encodes fucosyltransferase 2, an enzyme responsible for the secretion of fucosylated glycans on mucosal surfaces. These glycans serve as attachment sites and nutrient substrates for specific commensal bacteria. Individuals carrying loss-of-function FUT2 variants (“non-secretors”) exhibit altered gut microbiota composition, including reduced abundance of beneficial *Bifidobacterium* species. Since *Bifidobacteria* contribute to intestinal barrier integrity and SCFA production, FUT2 polymorphisms may indirectly affect susceptibility to dysbiosis, inflammation, and metabolic dysfunction [158].

Genes involved in microbial recognition pathways also play a major role in shaping microbiota composition. Nucleotide-binding oligomerization domain containing 2 (*NOD2*) is one of the best-characterized examples. *NOD2* encodes an intracellular pattern-recognition receptor (PRR) that detects bacterial peptidoglycan fragments and regulates immune responses toward intestinal bacteria [157]. Variants in *NOD2* may impair microbial recognition and alter immune tolerance, promoting dysbiosis and intestinal inflammation. Although *NOD2* polymorphisms are classically associated with inflammatory bowel disease, they also demonstrate the importance of host genetics in regulating microbial homeostasis.

Similarly, toll-like receptors, particularly TLR4, are involved in recognizing microbial components such as LPS from Gram-negative bacteria [89]. Altered TLR signaling may modify inflammatory responses and influence microbial composition by changing the intestinal immune environment. Excessive activation of these pathways may contribute to chronic low-grade inflammation and insulin resistance observed in MetS.

Host genetics may also regulate the production of antimicrobial peptides and mucus secretion. Genes controlling mucin synthesis influence the structure of the intestinal mucus layer, which serves as a protective barrier and ecological niche for gut microorganisms [159]. Alterations in mucus composition may therefore affect bacterial adhesion, colonization, and microbial diversity.

In addition to immune-related genes, metabolic genes associated with obesity and insulin resistance may indirectly influence microbiota composition. Polymorphisms in genes such as *FTO*, *PPARG*, *APOA5*, and *MC4R* may alter body weight regulation, adiposity, lipid metabolism, and inflammatory status, thereby modifying the intestinal microenvironment [13,66]. Variants in the *FTO* gene, which is strongly associated with obesity and appetite regulation, appear to influence microbial composition through alterations in dietary behavior and adiposity [66]. Individuals carrying *FTO* risk alleles frequently exhibit dietary patterns associated with dysbiosis, including increased intake of energy-dense foods and saturated fats. Similarly, polymorphisms in *PPARG*, *MC4R*, and *APOA5* may indirectly affect the intestinal microenvironment by altering lipid metabolism, adipose tissue function, and inflammatory signaling [154]. These metabolic alterations may favor the growth of pro-inflammatory microbial species and contribute to increased intestinal permeability and systemic inflammation.

Conversely, gut microbiota may regulate host metabolic pathways through microbial metabolites. SCFAs are among the most important mediators of microbiota–host communication and may influence the expression of genes involved in glucose metabolism, lipid homeostasis, inflammatory responses, appetite regulation, and intestinal barrier function [118,151]. Through these mechanisms, microbial metabolites may improve insulin sensitivity, reduce inflammatory cytokine production, and maintain intestinal barrier integrity. Dysbiosis-associated reductions in SCFA-producing bacteria may therefore contribute to altered gene expression and metabolic dysfunction.

Gene–microbiome interactions are also involved in the regulation of inflammatory pathways associated with MetS. LPS derived from Gram-negative bacteria can activate TLR4-mediated signaling, stimulating NF-κB activation and cytokine production [90]. The intensity of these inflammatory responses may vary according to host genetic polymorphisms affecting immune signaling pathways.

Recent GWAS and multi-omics approaches have further demonstrated associations between specific host genetic loci and microbial taxa abundance [97]. Certain bacterial groups, including *Christensenellaceae*, have been identified as highly heritable and associated with lower BMI, reduced visceral adiposity, and favorable metabolic profiles [98,154]. These findings support the concept that host genetic background may partly influence obesity-associated gut microbiota patterns.

Gene–microbiome interactions may also contribute to interindividual variability in metabolic responses to diet and therapeutic interventions. Individuals with similar dietary habits may exhibit different metabolic outcomes depending on both their genetic background and microbial composition [153]. This concept is particularly relevant for personalized medicine and precision nutrition, where preventive and therapeutic strategies may be tailored according to host genetic background, microbiome profile, metabolic phenotype, and dietary response.

Obesity-related metabolic changes may increase intestinal permeability and favor the growth of pro-inflammatory bacterial species.

Substantial interindividual variability exists in gut microbiota composition. Each individual possesses a unique microbial profile shaped by interactions between genetic background, diet, environment, medication exposure, and lifestyle factors [115]. Even among individuals living in similar environments, significant differences in microbial diversity and bacterial abundance can be observed.

Twin studies have provided strong evidence supporting the role of host genetics in microbiota variability. Comparisons between monozygotic and dizygotic twins demonstrated that monozygotic twins share more similar microbial profiles, suggesting a genetic contribution to microbial composition [160].

Interindividual differences in microbiota are clinically important because they influence nutrient metabolism, inflammatory responses, and energy extraction from the diet. Some individuals possess microbial communities that are more efficient at harvesting energy from indigestible carbohydrates, potentially increasing caloric availability and promoting fat accumulation [161]. This may partly explain why individuals consuming similar diets may exhibit different susceptibility to obesity and MetS.

Variability in microbiota composition may additionally influence responses to dietary interventions and pharmacological treatments. For example, differences in microbial metabolism can affect the production of SCFAs, bile acid transformation, and gut hormone secretion, all of which contribute to glucose homeostasis and appetite regulation [118]. Consequently, individuals with distinct microbiota profiles may respond differently to the same dietary or therapeutic intervention.

The interaction between host genetics and microbiota is also bidirectional. While host DNA shapes the intestinal microbial environment, gut microorganisms may influence host gene expression through epigenetic mechanisms. Microbial metabolites such as butyrate can regulate histone acetylation and DNA methylation, thereby affecting metabolic and inflammatory pathways [151]. This complex interaction contributes to the regulation of energy metabolism, immune homeostasis, and susceptibility to metabolic diseases.

Epigenetic regulation provides an additional mechanism through which microbiota-derived signals may influence host metabolism and inflammatory responses.

Unlike genetic mutations, epigenetic modifications do not alter the DNA nucleotide sequence itself but regulate gene expression through reversible chemical changes affecting chromatin structure and transcriptional activity [162]. These mechanisms include DNA methylation, histone modifications, and non-coding RNA regulation, all of which may be influenced by diet, physical activity, stress, smoking, sleep patterns, and gut microbiota composition. Increasing evidence suggests that epigenetic alterations contribute to obesity, insulin resistance, chronic low-grade inflammation, dyslipidemia, and type 2 diabetes mellitus, which are major components of MetS [163]. Furthermore, epigenetic modifications are dynamic and potentially reversible, making them attractive targets for preventive and therapeutic strategies.

DNA methylation is one of the most extensively studied epigenetic mechanisms in metabolic diseases. It involves the addition of a methyl group to cytosine residues within CpG dinucleotides through the action of DNA methyltransferases (DNMTs) [164]. In most cases, methylation within promoter regions suppresses gene transcription by limiting the accessibility of transcription factors to DNA.

Altered DNA methylation patterns have been identified in multiple genes involved in glucose metabolism, lipid homeostasis, inflammation, and adipogenesis in individuals with obesity and MetS [165]. For example, abnormal methylation of the *PPARG* gene, a key regulator of adipocyte differentiation and insulin sensitivity, has been associated with obesity and insulin resistance [166]. Similarly, methylation changes in genes involved in insulin signaling pathways and inflammatory responses may contribute to impaired glucose homeostasis and chronic inflammation.

Recent studies also suggest that obesity-related metabolic disturbances are associated with tissue-specific epigenetic changes. Epigenetic alterations have been described in adipose tissue, liver, skeletal muscle, and pancreatic β-cells, all of which are central organs in metabolic regulation [167]. In individuals with obesity, differential methylation patterns in adipose tissue are associated with altered expression of genes involved in lipid metabolism, inflammatory signaling, and mitochondrial function.

DNA methylation is strongly influenced by environmental and nutritional factors. Nutrients involved in one-carbon metabolism, such as folate, vitamin B12, methionine, and choline, are essential for methyl donor availability and may therefore directly affect methylation processes [168]. Deficiencies or imbalances in these nutrients may alter epigenetic regulation and contribute to metabolic dysfunction.

Importantly, epigenetic programming may begin early in life. Maternal obesity, gestational diabetes, and prenatal nutritional status may induce long-lasting epigenetic changes that predispose offspring to obesity and metabolic syndrome in adulthood [169]. This concept supports the developmental origins of health and disease (DOHaD) hypothesis, according to which early-life environmental exposures influence long-term metabolic risk.

Diet and lifestyle are major modulators of epigenetic regulation and metabolic health. Western dietary patterns characterized by high intake of saturated fats, refined sugars, and ultra-processed foods have been associated with pro-inflammatory gene expression profiles and increased metabolic disease risk [83]. In contrast, Mediterranean-style diets rich in fruits, vegetables, dietary fiber, omega-3 fatty acids, and polyphenols appear to exert protective epigenetic effects.

Several bioactive food compounds can modulate epigenetic pathways. Polyphenols such as resveratrol, curcumin, quercetin, and epigallocatechin gallate (EGCG) may regulate DNA methylation and histone acetylation, thereby influencing the expression of genes involved in oxidative stress, inflammation, and insulin sensitivity [170]. Omega-3 fatty acids additionally modulate transcription factors associated with lipid metabolism and inflammatory pathways.

Physical activity also exerts beneficial epigenetic effects. Exercise has been associated with methylation changes in genes regulating glucose transport, mitochondrial biogenesis, and insulin signaling [171]. These adaptations may contribute to improved insulin sensitivity and reduced cardiovascular risk in physically active individuals.

Conversely, unhealthy lifestyle factors such as smoking, chronic psychological stress, sedentary behavior, and sleep deprivation may induce adverse epigenetic modifications associated with chronic inflammation and metabolic dysfunction [172]. Chronic stress may alter HPA axis regulation through epigenetic modulation of glucocorticoid receptor genes, thereby affecting cortisol secretion and energy homeostasis.

Recent evidence additionally suggests that paternal lifestyle factors may influence offspring metabolic health through epigenetic mechanisms. Paternal obesity and poor dietary habits have been associated with altered sperm epigenetic profiles linked to metabolic disturbances in offspring [173]. These findings highlight the transgenerational impact of environmental exposures on metabolic disease susceptibility.

The gut microbiota has emerged as an important regulator of host epigenetic mechanisms. Gut microorganisms produce a wide range of metabolites capable of modulating gene expression and epigenetic pathways [174]. Among these metabolites, SCFAs, particularly butyrate, acetate, and propionate, play central roles in microbiota–host interactions. Butyrate is particularly important because it acts as a Histone Deacetylase (HDAC) inhibitor [175]. Histone acetylation regulates chromatin accessibility and gene transcription. By inhibiting HDAC activity, butyrate promotes the transcription of genes involved in anti-inflammatory responses, intestinal barrier integrity, and metabolic homeostasis. Reduced abundance of butyrate-producing bacteria in dysbiosis may therefore contribute to impaired epigenetic regulation and metabolic dysfunction.

Recent multi-omics studies have demonstrated strong associations between gut microbiota composition and DNA methylation profiles in individuals with obesity, insulin resistance, and type 2 diabetes [152]. Specific bacterial taxa, including *Odoribacteriaceae*, *Christensenellaceae*, and *Faecalibacterium*, have been associated with methylation changes in genes involved in lipid metabolism, inflammatory signaling, and glucose homeostasis. These findings suggest that gut microbiota may directly influence host epigenetic regulation in metabolic diseases.

Microbial metabolites may also influence methyl donor pathways. Certain intestinal bacteria participate in folate and B-vitamin metabolism, thereby affecting the availability of substrates required for DNA methylation [95]. Consequently, alterations in microbiota composition may indirectly modify host methylation patterns and gene expression. Thus, microbial metabolites act as biochemical intermediates between host genetic susceptibility, microbiota composition, and metabolic phenotype. For example, reduced butyrate availability may impair HDAC-related epigenetic regulation and barrier protection, whereas increased LPS signaling may interact with genetically determined immune responsiveness to amplify inflammatory and metabolic dysfunction.

The interaction between microbiota and epigenetic regulation is therefore dynamic and reciprocal. While microbial metabolites can regulate host epigenetic pathways, host epigenetic mechanisms may also affect immune responses, intestinal permeability, and microbial composition [96]. Together, DNA methylation, lifestyle-related epigenetic modifications, and microbiota-derived metabolites influence inflammation, insulin sensitivity, and energy metabolism, thereby linking environmental exposures, microbial activity, and genetic susceptibility in MetS. Taken together, gene–microbiome interactions provide a mechanistic link between inherited susceptibility, microbial ecology, inflammatory activation, epigenetic regulation, and metabolic dysfunction. These interactions may help explain why individuals with similar environmental exposures develop different metabolic phenotypes and why responses to dietary or microbiota-targeted interventions are highly variable. Understanding these mechanisms may support personalized strategies targeting both host-related and microbiota-related pathways in MetS.

Experimental, clinical, genetic, and multi-omics evidence supporting the role of host genetics, epigenetic regulation, and gene–microbiome interactions in MetS is summarized in Table 3. The table distinguishes human genetic association studies, preclinical evidence, and multi-omics/epigenetic evidence, while also indicating the microbiota-related taxa, metabolites, or pathways involved.

### 4.4. Integrated Microbiota–Gut–Brain–Metabolic Model of MetS Pathogenesis

Although the mechanisms connecting gut dysbiosis with MetS have been discussed separately, they are better understood as interacting elements of a coordinated pathophysiological network rather than as independent processes. Within this integrative framework, dietary imbalance, sedentary behavior, genetic susceptibility, medication exposure, stress, and circadian disruption may reshape gut microbiota composition and reduce microbial diversity [66,69,101,102,103,104,105,106,107,108]. This dysbiotic profile may be accompanied by depletion of beneficial SCFA-producing bacteria, disturbances in bile acid metabolism, increased generation of pro-inflammatory microbial products, and impairment of intestinal barrier integrity [67,71,77,80,88,104].

Within this model, several biochemical pathways appear particularly relevant: SCFA–FFAR2/3/GPR109A signaling, LPS–TLR4/NF-κB-mediated inflammatory activation, bile acid–FXR/TGR5 signaling, TMA–TMAO metabolism, tryptophan–indole–AhR signaling, enteroendocrine GLP-1/PYY secretion, vagal signaling, HPA axis activation, and microbiota-related epigenetic regulation [73,74,75,76,77,82,84,85,86,89,90,91,92,93,94].

An important early step in this process is impairment of the intestinal epithelial barrier, which increases intestinal permeability and facilitates the translocation of bacterial components, particularly LPS, into the systemic circulation. Circulating LPS activates TLR4/NF-κB-dependent inflammatory signaling in immune cells, adipose tissue, the liver, and skeletal muscle, thereby promoting chronic low-grade inflammation [64,89,90]. This inflammatory milieu disrupts insulin signaling through cytokine-mediated impairment of the IRS-1/PI3K/Akt pathway, contributing to insulin resistance, reduced peripheral glucose uptake, and increased hepatic glucose production [22,23,24,25,26,62,63].

In parallel, dysbiosis alters the production of microbial metabolites involved in metabolic and neuroendocrine regulation. Reduced SCFA availability may compromise intestinal barrier integrity, decrease GLP-1 and PYY secretion, disturb appetite control, and attenuate anti-inflammatory signaling [73,81,117]. Changes in bile acid metabolism may further impair FXR- and TGR5-mediated regulation of glucose and lipid homeostasis, whereas increased TMAO production may contribute to endothelial dysfunction and cardiovascular risk [74,76,77,88,100]. Together, these metabolic alterations may promote dyslipidemia, visceral adiposity, and vascular dysfunction, all of which are key components of MetS [31,32,33,36,37].

The gut–brain axis further integrates these processes by linking microbial metabolites, inflammatory mediators, gut-derived hormones, and vagal signaling with central pathways involved in appetite, satiety, stress responses, autonomic regulation, and energy expenditure [10,11,12,13,81,117,119,120]. Conversely, chronic stress and HPA axis activation may affect intestinal permeability, immune activity, and microbiota composition, creating a bidirectional feedback loop between neuroendocrine dysregulation and metabolic impairment [50,51,52,53,54,108].

Host genetics and epigenetic regulation may modulate several components of this network. Variants affecting immunity, metabolism, intestinal barrier function, and microbial colonization can influence susceptibility to dysbiosis and metabolic dysfunction [97,98,153,154]. At the same time, microbiota-derived metabolites may regulate host gene expression through epigenetic mechanisms such as DNA methylation and histone modifications [151,152]. Thus, the transition from dysbiosis to MetS reflects a dynamic interplay between microbial, inflammatory, metabolic, neuroendocrine, genetic, and epigenetic pathways.

These interconnected mechanisms suggest that gut dysbiosis may promote MetS through a self-reinforcing cycle involving intestinal barrier dysfunction, metabolic endotoxemia, chronic inflammation, altered microbial metabolite production, impaired insulin signaling, disrupted lipid metabolism, and altered appetite regulation. Visceral adiposity and metabolic dysfunction may further sustain this cycle by amplifying inflammation and perpetuating microbiota disruption [29,64,73,77,89,90]. This integrated framework helps explain the systemic nature of MetS and supports the development of personalized, mechanism-based strategies targeting both host and microbial pathways [97,151,152,153].

## 5. Personalized Microbiota-Based and Multi-Omics Approaches in MetS

### 5.1. Rationale for Personalized Medicine in MetS

The growing understanding of the interactions between the gut microbiota, the gut–brain axis, host genetics, epigenetic regulation, and metabolic pathways has important implications for personalized approaches to the prevention and management of MetS. Traditionally, MetS management has focused mainly on treating individual components such as obesity, dyslipidemia, hypertension, and hyperglycemia. However, recent evidence suggests that MetS should be approached as a multifactorial and biologically heterogeneous disorder involving complex interactions between metabolic, inflammatory, microbial, genetic, and neuroendocrine pathways [66].

The concept of personalized medicine is particularly relevant in MetS because individuals with similar clinical phenotypes may differ substantially in genetic susceptibility, gut microbiota composition, inflammatory profile, epigenetic regulation, lifestyle exposures, and response to therapy [97,151,152,153]. Therefore, a uniform therapeutic approach may not adequately address the biological heterogeneity underlying MetS. In this context, integration of host-related factors and microbiota-derived information may allow more precise patient stratification and the identification of targeted preventive and therapeutic strategies.

One of the most important clinical implications of these findings is the increasing recognition of the gut microbiota as a potential therapeutic target. Dietary interventions capable of modulating microbial composition may improve metabolic homeostasis and reduce systemic inflammation. Mediterranean-style diets rich in dietary fiber, polyphenols, fruits, vegetables, legumes, and omega-3 fatty acids have consistently been associated with increased microbial diversity, enhanced SCFA production, and improved insulin sensitivity [83]. In contrast, Western dietary patterns rich in saturated fats and ultra-processed foods promote dysbiosis, intestinal permeability, and chronic inflammation.

### 5.2. Microbiota-Targeted and Personalized Therapeutic Approaches

Probiotics and prebiotics have also emerged as promising therapeutic approaches. Several studies suggest that supplementation with beneficial bacterial strains such as *Bifidobacterium* and *Lactobacillus* may improve glucose metabolism, reduce inflammatory markers, and support intestinal barrier integrity [180]. Prebiotics, including inulin and fructooligosaccharides, stimulate the growth of SCFA-producing bacteria and may contribute to improved metabolic outcomes. Nevertheless, the clinical efficacy of probiotic interventions remains variable, partly because of interindividual differences in genetics, baseline microbiota composition, diet, and environmental exposures. This heterogeneity supports the need for personalized microbiota-targeted strategies rather than a universal probiotic or prebiotic approach. Future interventions may involve selecting bacterial strains, prebiotic substrates, or synbiotic combinations according to the patient’s baseline microbiome profile, dietary background, and dominant metabolic alteration.

Fecal microbiota transplantation (FMT) has attracted increasing interest as a potential therapy for metabolic disorders. Experimental and clinical studies have demonstrated that transplantation of microbiota from metabolically healthy donors may temporarily improve insulin sensitivity in individuals with obesity and MetS [181]. However, the long-term efficacy, safety, donor selection criteria, and standardization of FMT protocols remain important challenges requiring further investigation. In a personalized medicine framework, FMT would require careful donor selection, recipient stratification, safety evaluation, and standardized protocols before it can be considered for routine management of MetS.

The identification of gene–microbiome interactions has also contributed to the development of personalized medicine approaches. Individuals carrying specific genetic polymorphisms may respond differently to dietary interventions, pharmacological treatments, or microbiota-targeted therapies [97]. For example, variants in genes associated with obesity, insulin resistance, inflammation, and lipid metabolism may influence microbiota composition and metabolic responses to nutrients. Consequently, integrating genetic, microbial, and metabolic profiling may improve risk stratification and allow the development of individualized preventive strategies. Multi-omics approaches, combining genomics, epigenomics, metagenomics, metabolomics, transcriptomics, and clinical data, may improve patient stratification beyond conventional diagnostic criteria [97,152,153,154,182]. Individuals with MetS may differ in microbial metabolite profiles, including SCFAs, bile acid derivatives, TMAO, and LPS-related inflammatory markers [76,77,81,118]. These differences may help identify subgroups of patients in whom the predominant mechanism is dysbiosis-driven inflammation, impaired microbial metabolite production, altered bile acid signaling, or gene–microbiome interaction.

Precision nutrition represents one of the most promising and clinically applicable directions in personalized metabolic medicine. Dietary responses are highly variable among individuals and may depend on baseline microbiota composition, host genetic background, insulin sensitivity, medication use, age, lifestyle, and glycemic response profiles [69,111,153,154]. Personalized dietary interventions based on genetic background, gut microbiota composition, and metabolic response may improve treatment efficacy and reduce metabolic risk.

Epigenetic mechanisms also provide important therapeutic perspectives. Since epigenetic modifications are potentially reversible, interventions targeting DNA methylation, histone modifications, and microbiota-derived metabolites may contribute to metabolic disease prevention and treatment. Lifestyle modifications, including regular physical activity, healthy dietary patterns, stress reduction, and sleep optimization, may exert beneficial epigenetic effects and improve metabolic regulation.

### 5.3. Challenges and Future Perspectives

Despite its promise, personalized medicine in MetS faces several challenges. The gut microbiota is highly dynamic and influenced by numerous confounding factors, including geography, ethnicity, age, medication use, circadian rhythm, lifestyle, and dietary habits [101,107,115]. In addition, microbiome sequencing, metabolite measurement, and bioinformatic analysis are not yet standardized across studies, limiting reproducibility and clinical translation [115,153]. Many current studies are observational, making it difficult to establish causal relationships between dysbiosis and metabolic disease.

Another major challenge is the substantial interindividual variability in microbiota composition and metabolic responses. Two individuals exposed to similar diets may exhibit different metabolic outcomes due to differences in genetic background, microbial composition, immune regulation, epigenetic profile, and environmental exposures [153]. This complexity highlights the need for integrative and personalized approaches rather than universal therapeutic strategies.

Future research should therefore focus on large-scale longitudinal studies and well-designed clinical trials integrating genetic, epigenetic, microbial, metabolomic, lifestyle, and clinical data. Improved understanding of the gut–brain axis and gene–microbiome interactions may contribute to the identification of novel biomarkers and therapeutic targets for MetS [117]. Furthermore, advances in artificial intelligence and machine learning may facilitate the development of predictive models capable of identifying individuals at high metabolic risk and optimizing personalized interventions. However, such models require validation in large, diverse, and independent cohorts before routine clinical implementation.

Overall, the integration of microbiome science, genetics, epigenetics, and metabolic medicine has transformed current perspectives on MetS. These emerging insights support the transition from conventional symptom-based management toward personalized and mechanism-based therapeutic strategies targeting both host and microbial pathways.

## 6. Strengths and Limitations of the Review

This review provides a multidisciplinary overview of MetS by integrating the roles of the gut microbiota, GBA, genetics, and epigenetic regulation in metabolic dysfunction. An important strength of the article is the comprehensive presentation of the pathophysiological mechanisms linking dysbiosis, chronic inflammation, insulin resistance, and metabolic alterations. In addition, the review includes recent experimental and clinical evidence regarding gene–microbiome interactions and highlights the growing importance of personalized medicine and precision nutrition in MetS management.

However, several limitations should also be acknowledged. Many of the available studies are observational or experimental, which limits the ability to establish definitive causal relationships between microbiota alterations and MetS. Furthermore, significant heterogeneity exists among microbiome studies due to differences in study populations, dietary patterns, sequencing techniques, and analytical methodologies. The gut microbiota is also highly dynamic and influenced by multiple environmental and lifestyle-related factors, making comparisons between studies difficult. In addition, microbiota-targeted therapeutic approaches, including probiotics and FMT, still lack standardized protocols and sufficient long-term clinical evidence. Finally, substantial interindividual variability in both genetic background and microbiota composition may limit the generalizability of current findings.

## 7. Conclusions

The GBA plays an important role in the development and progression of MetS through complex interactions involving the gut microbiota, immune system, endocrine signaling, and host genetics. Dysbiosis contributes to insulin resistance, chronic low-grade inflammation, altered intestinal permeability, and metabolic dysfunction, highlighting the central role of the microbiome in metabolic homeostasis.

Genetic susceptibility, epigenetic regulation, and environmental factors such as diet and lifestyle further influence microbiota composition and interindividual metabolic responses. These interactions emphasize the multifactorial nature of MetS and support the concept of personalized medicine.

Emerging therapeutic strategies targeting the gut microbiota, including dietary interventions, probiotics, prebiotics, and microbiome-based personalized approaches, may improve metabolic outcomes. However, further longitudinal and mechanistic studies are needed to better understand gene–microbiome interactions and their clinical applications in MetS.

## Figures and Tables

**Table 1:** Representative gut bacteria, microbial metabolites, and biochemical pathways linking gut dysbiosis, gut–brain axis signaling, and metabolic syndrome.

Bacterial Taxa/Microbial Group	Main Metabolites or Microbial Products	Main Biochemical Pathway	Relevance for Gut–Brain Axis and MetS	References
*Faecalibacterium prausnitzii* and *Roseburia* spp.	Butyrate and other SCFAs	Activation of FFAR2/FFAR3 and GPR109A signaling; reinforcement of tight junctions; Treg induction; inhibition of NF-κB-mediated inflammation; HDAC inhibition	Supports intestinal barrier integrity, reduces low-grade inflammation, improves insulin sensitivity and may influence GLP-1/PYY secretion, appetite regulation, and gut–brain communication	Rinnella et al., 2019 [67] Canfora et al., 2019 [73] Dalile et al., 2019 [81] Yang et al., 2020 [82]
*Bifidobacterium* spp.	Acetate, lactate, SCFA cross-feeding products	Enhancement of epithelial barrier function; immune tolerance modulation; support of SCFA-producing microbial networks	May reduce metabolic endotoxemia, support gut hormone secretion, improve glucose homeostasis, and contribute to more favorable metabolic profiles	Rinnella et al., 2019 [67] Blaak et al., 2020 [71] Zinocker et al., 2018 [83]
*Lactobacillus* spp.	Lactate, GABA-related and tryptophan-related metabolites	Modulation of enteroendocrine signaling, immune responses, and neurotransmitter-related pathways; possible effects on GABAergic and serotonergic signaling	May influence appetite regulation, stress-related responses, gut barrier function, and inflammatory tone within the gut–brain axis	Loh et al., 2024 [84] Wei et al., 2022 [85] Lui et al., 2022 [86]
*Akkermansia muciniphila*	Mucin-derived metabolites, acetate, propionate-related pathways	Regulation of mucus layer integrity; improvement of epithelial barrier function; reduction in LPS translocation and metabolic endotoxemia	Inversely associated with obesity, insulin resistance, and MetS; may reduce inflammation and improve metabolic phenotype	Cani et al., 2018 [29,87] Depommier et al., 2019 [80]
*Bacteroides* spp. and *Prevotella* spp.	Propionate, succinate, bile acid-modifying enzymes	Fermentation of complex carbohydrates; modulation of bile acid pools; activation of FFAR2/3 and FXR/TGR5-related pathways	May influence satiety, hepatic glucose synthesis, lipid metabolism, and energy homeostasis; effects may depend on diet and microbial context	Blaak et al., 2020 [71] Wahlstrom et al., 2016 [74] Collins et al., 2023 [75]
*Clostridial clusters IV* and *XIVa*	Butyrate and secondary bile acid-related metabolites	Treg differentiation; anti-inflammatory signaling; bile acid transformation; epithelial barrier support	Contribute to immune tolerance, intestinal barrier maintenance, and regulation of metabolic inflammation	Belkaid et al., 2014 [78] Thaiss et al., 2016 [79] Yang et al., 2020 [82]
*Bilophila wadsworthia* and bile-tolerant bacteria	Hydrogen sulfide and pro-inflammatory microbial products	Expansion under high-fat diet conditions; bile acid-dependent growth; epithelial barrier disruption	May promote intestinal inflammation, increased permeability, metabolic endotoxemia, and dysbiosis-associated metabolic dysfunction	Collins et al., 2023 [75] Devkota et al., 2012 [88]
*Proteobacteria*/ *Enterobacteriaceae*	LPS and other pathogen-associated molecular patterns	Activation of TLR4/MyD88/NF-κB and MAPK signaling; increased cytokine production	Promotes chronic low-grade inflammation, adipose tissue dysfunction, insulin resistance, endothelial dysfunction, and MetS progression	Cani et al., 2007 [64] Rohr et al., 2020 [89] Saltiel et al., 2017 [90]
TMA-producing bacteria, including selected *Clostridium* spp. and *Enterobacteriaceae*	Trimethylamine, converted in the liver to TMAO	Microbial choline/carnitine metabolism; hepatic FMO3-dependent TMAO formation; endothelial and inflammatory signaling	Associated with atherosclerosis, endothelial dysfunction, cardiovascular risk, and MetS progression	Wang et al., 2011 [76] Janeiro et al., 2018 [77]
Tryptophan-metabolizing bacteria, including *Lactobacillus* spp., *Bifidobacterium* spp., and indole-producing taxa	Indole derivatives, serotonin-modulating metabolites, kynurenine-related metabolites	AhR signaling; serotonin synthesis modulation; immune and neuroendocrine regulation	May influence intestinal barrier integrity, inflammation, mood, appetite regulation, HPA axis activity, and gut–brain signaling	Loh et al., 2024 [84] Lui et al., 2022 [86] Lorsch et al., 2026 [91] Wolfson et al., 2023 [92] Ravisankar et al., 2026 [93] Zheng et al., 2026 [94]
Folate- and B-vitamin-producing bacteria	Folate and B vitamins involved in one-carbon metabolism	Regulation of methyl donor availability; DNA methylation and gene expression	May connect gut microbiota composition with epigenetic regulation, metabolic inflammation, and interindividual variability in MetS	Rowland et al., 2018 [95] Qiu et al., 2018 [96]
Heritable taxa, including *Christensenellaceae*	Taxon-specific metabolic profiles; SCFA-related and host-associated metabolic effects	Host genetic modulation of microbial abundance; gene–microbiome interaction; multi-omics associations	Associated with lower BMI and favorable metabolic profiles; may partly explain interindividual differences in obesity-related microbiota patterns	Kurilshikov et al., 2021 [97] Waters et al., 2019 [98]

**Table 2:** Lifestyle factors, metabolic syndrome, and selected microbiota-related findings.

Lifestyle Exposure/ Intervention	Study Type and Population	Microbiota-Related Findings	Metabolic/Clinical Relevance	Critical Appraisal and Limitations	Reference
**Observational clinical studies**					
Western dietary pattern	Observational cohort, multi-omics, adults	Associated with lower gut microbial diversity and altered microbiota-related inflammatory profiles	Associated with reduced insulin sensitivity and may increase MetS risk through microbiota-related inflammatory and metabolic pathways	Observational design limits causal interpretation; residual confounding from diet, lifestyle, medication use, and baseline metabolic risk remains possible	Asnicar et al., 2021 [69]
Healthy dietary pattern rich in fiber, fruits, and vegetables	Prospective cohort, 6213 adults	Associated with potentially beneficial microbiota composition and support of fiber-fermenting bacterial communities	Associated with approximately 20–30% lower MetS risk; may improve metabolic profile through SCFA-related mechanisms	Dietary exposure may be affected by self-reporting bias and unmeasured confounding factors	Kim et al., 2025 [111]
Smoking	Prospective cohort, 6213 adults	Smoking may negatively affect gut microbial balance and promote pro-inflammatory microbial patterns	Current smokers showed higher MetS risk than non-smoke	Microbiota changes may be influenced by diet, alcohol intake, physical activity, and socioeconomic factors	Kim et al., 2025 [111]
Smoking	Clinical observational study	Associated with altered microbiota composition and increased systemic inflammation	May contribute to metabolic dysfunction through microbiota-related inflammatory pathways	Causality and direct links to MetS outcomes require longitudinal confirmation	Biedermann et al., 2023 [109]
Alcohol consumption	Prospective cohort, 6213 adults	Heavy alcohol intake may impair gut microbiota composition and intestinal barrier integrity	Moderate intake showed neutral or mildly protective associations, whereas heavy intake increased MetS risk	Differences in alcohol dose, drinking patterns, and lifestyle factors may limit interpretation	Kim et al., 2025 [111]
**Reviews and meta-analyses**					
High-fat, high-sugar dietary pattern	Narrative review	Associated with gut microbiota dysbiosis, reduced microbial diversity, altered bile acid metabolism, and reduced SCFA production	May contribute to dyslipidemia, insulin resistance, inflammation, and MetS development	Narrative synthesis; findings should be interpreted qualitatively, as no direct effect-size estimate is provided	Hamooya et al., 2025 [34]
Smoking	Meta-analysis of prospective studies	Smoking may contribute to metabolic risk partly through inflammatory and microbiota-related mechanisms	Associated with higher risk of MetS and central obesity	Variability in MetS definitions, exposure assessment, and confounder adjustment may affect comparability between studies	Sun et al., 2012 [112]
Fruits, vegetables, whole grains, and processed meat intake	Systematic review and meta-analysis	Fiber-rich diets may support beneficial SCFA-producing bacteria, whereas processed meat intake may favor unfavorable microbial and inflammatory profiles	Higher intake of fruits, vegetables, and whole grains was associated with lower MetS risk, whereas processed meat intake was associated with higher risk	Variability in dietary patterns and reliance on observational data limit causal interpretation	Schwingshackl et al., 2017 [113]
**Interventional evidence**					
Alcohol reduction and lifestyle modification	Lifestyle intervention study	May favor partial restoration of microbiota balance and improved microbial metabolic activity	Improved metabolic parameters and may reduce cardiometabolic risk	Reproducibility may be limited by sample size, intervention duration, adherence, and incomplete microbiota assessment	Powell et al., 2026 [114]

**Table 3:** Experimental and clinical studies investigating gene–microbiome interactions in metabolic syndrome.

Genetic/Epigenetic Mechanism	Evidence Type and Model/Population	Microbiota Taxa, Metabolites, or Pathways Involved	Main Finding and Metabolic Relevance	Critical Appraisal and Limitations	Reference
**Human genetic association evidence**					
FUT2 polymorphisms and mucosal microbial colonization	Human genetic association study	Reduced *Bifidobacterium* abundance; altered mucosal microbiota; glycan-dependent host–microbe interactions	The FUT2 non-secretor genotype was associated with altered mucosal microbiota composition, suggesting that host glycosylation-related genes may influence microbial colonization and susceptibility to dysbiosis-related metabolic dysfunction	Population characteristics, disease background, environmental exposures, and microbiota assessment methods may influence the results; causal interpretation remains limited	Rausch et al. [158]
Heritable bacterial taxa associated with obesity	Twin study	*Christensenellaceae* and other heritable microbial taxa	*Christensenellaceae* were identified as highly heritable and associated with lower BMI, suggesting that host genetic background may partly influence obesity-associated gut microbiota patterns	Shared environment, diet, lifestyle, and non-genetic factors may also contribute to the observed associations	Goodrich et al. [155]
Host genetic loci associated with microbiota variability	Large-scale GWAS, over 18,000 individuals	Multiple microbial taxa and microbiota-related metabolic pathways	Several host genetic loci were associated with microbial abundance and metabolic pathway variation, supporting a role for host genetics in interindividual microbiome variability relevant to metabolic disease	Reported genetic effect sizes are generally small; population heterogeneity and microbiome assessment methods may limit reproducibility; functional validation is needed	Kurilshikov et al. [97]
Genetic determinants of microbial composition	Population-based cohort study	Immune- and metabolism-related microbial taxa	Variants in immune and metabolic genes were associated with the abundance of specific gut bacteria, suggesting interactions between host genetic background, immune-metabolic regulation, and microbial composition	Associations may be influenced by diet, medication use, geography, environmental exposures, and methodological differences in microbiome profiling	Bonder et al. [176]
Obesity-associated genetic variants and microbiota	Human cohort study	Altered microbial richness and obesity-related microbiota patterns	Obesity-risk alleles were associated with changes in microbial richness and metabolic parameters, suggesting that genetic susceptibility may contribute to metabolic dysfunction partly through microbiota-related mechanisms	The relationship may be indirect and influenced by adiposity, diet, medication use, lifestyle, and environmental factors	Turpin et al. [177]
**Preclinical and experimental evidence**					
NOD2 variants and microbiota-mediated inflammation	Experimental mouse study	Dysbiosis; immune-related host–microbiota interaction; inflammatory signaling	NOD2-deficient mice developed dysbiosis, inflammation, and insulin resistance under high-fat diet conditions, suggesting that immune-related genes may influence microbiota composition and metabolic phenotype	Mouse models provide mechanistic insight, but differences in diet, immune regulation, microbiota composition, and host genetics limit direct translation to human MetS	Denou et al. [178]
TLR5 deficiency and microbiota-induced metabolic syndrome	Experimental mouse study	Altered gut microbiota; innate immune signaling; microbiota-dependent metabolic inflammation	TLR5-deficient mice developed hyperphagia, obesity, insulin resistance, and altered gut microbiota, indicating that impaired innate immune signaling may promote MetS-like features through microbiota-dependent pathways	Animal models provide strong mechanistic support, but human relevance remains to be confirmed in longitudinal and clinical studies	Vijay-Kumar et al. [179]
**Multi-omics and epigenetic evidence**					
Microbiome-associated DNA methylation patterns	Human multi-omics metabolic disease study	*Odoribacteriaceae*, *Christensenellaceae*, *Faecalibacterium*; DNA methylation; lipid and glucose metabolism pathways	Specific microbial taxa were associated with DNA methylation changes in genes involved in insulin resistance, lipid metabolism, and inflammatory signaling, supporting a potential role for microbiota-related epigenetic regulation in MetS	Multi-omics studies provide integrative insight, but findings are largely associative and the directionality between microbiota changes and epigenetic modifications remains uncertain	Martínez-Montoro et al. [152]
SCFAs and epigenetic regulation of host genes	Experimental and mechanistic study	SCFAs, especially butyrate; histone acetylation; HDAC inhibition	Microbial metabolites regulated histone acetylation and metabolic gene expression, suggesting that SCFAs may link microbiota activity with host epigenetic regulation and metabolic dysfunction	Mechanistic evidence supports biological plausibility, but clinical relevance requires validation in human metabolic studies with standardized metabolite assessment	Krautkramer et al. [151]

## Data Availability

No new data were created or analyzed in this study. Data sharing is not applicable to this article.

## Abbreviations

5-HT	5-hydroxytryptamine (serotonin)
11β-HSD1	11β-hydroxysteroid dehydrogenase type 1
ACTH	adrenocorticotropic hormone
ADIPOQ	adiponectin gene
Akt	protein kinase B
AMP	adenosine monophosphate
APOA5	apolipoprotein A5 gene
ApoB/ApoC-III	apolipoprotein B/apolipoprotein C-III
ATP	adenosine triphosphate
BBB	blood–brain barrier
BDNF	brain-derived neurotrophic factor
BMI	body mass index
CCK	cholecystokinin
CETP	cholesteryl ester transfer protein
CNS	central nervous system
CRH	corticotropin-releasing hormone
CRP	C-reactive protein
DAMPs	danger-associated molecular patterns
DNA	deoxyribonucleic acid
DNMTs	DNA methyltransferases
DOHaD	developmental origins of health and disease
EECs	enteroendocrine cells
EGCG	epigallocatechin gallate
ENS	enteric nervous system
FFAs	free fatty acids
FFAR2/FFAR3	free fatty acid receptor 2/free fatty acid receptor 3
FOXO1	forkhead box protein O1
FMT	fecal microbiota transplantation
FTO	fat mass and obesity-associated gene
FUT2	fucosyltransferase 2
FXR	farnesoid X receptor
GABA	γ-aminobutyric acid
GBA	gut–brain axis
GBPAR1	G protein-coupled bile acid receptor 1
GIP	glucose-dependent insulinotropic polypeptide
GLP-1	glucagon-like peptide-1
GPR41/GPR43/GPR109A	G-protein-coupled receptors 41/G-protein-coupled receptors 43/G-protein-coupled receptors 109A
GSK-3	glycogen synthase kinase-3
GWAS	genome-wide association studies
HDAC	histone deacetylase
HDL-C	HDL-cholesterol
HIF-1α	hypoxia-inducible factor-1α
HPA	hypothalamic–pituitary–adrenal
IFN-γ	interferon-gamma
IgA	immunoglobulin A
IL-6/IL-1β/IL-17/IL-18	interleukin-6/interleukin-1β/interleukin-17/interleukin-18
IR	insulin receptor
IRS-1	insulin receptor substrate-1
JNK	c-Jun N-terminal kinase
LDL-C	low-density lipoprotein cholesterol
LPL	lipoprotein lipase
LPS	lipopolysaccharide
MAPK	mitogen-activated protein kinase
MASLD	metabolic dysfunction-associated steatotic liver disease
MC4R	melanocortin-4 receptor gene
MCP-1	monocyte chemoattractant protein-1
MeSH	medical subject headings
MetS	metabolic syndrome
mTOR	mechanistic target of rapamycin
NAFLD	non-alcoholic fatty liver disease
NF-κB	nuclear factor kappa B
NO	nitric oxide
NLRs	NOD-like receptors
NLRP3	NOD-like receptor family pyrin domain-containing 3
NOD2	nucleotide-binding oligomerization domain containing 2
NTS	nucleus tractus solitarius
PAMPs	pathogen-associated molecular patterns
PCOS	polycystic ovary syndrome
PI3K	phosphatidylinositol-3-kinase
PIP2	phosphatidylinositol-4,5-bisphosphate
PIP3	phosphatidylinositol-3,4,5-trisphosphate
PKC	protein kinase C
PPARG	peroxisome proliferator-activated receptor gamma
PRR	pattern-recognition receptor
PYY	peptide YY
RNA	ribonucleic acid
ROS	reactive oxygen species
S6K	ribosomal protein s6 kinase
SCFAs	short-chain fatty acids
SGLT1	sodium-glucose cotransporter 1
TCF7L2	transcription factor 7-like 2
TGR5	Takeda G protein-coupled receptor 5
Th17	T helper 17 cells
TLR-4	toll-like receptor 4
TMA	trimethylamine
TMAO	trimethylamine N-oxide
TNF-α	tumor necrosis factor α
Tregs	regulatory T cells
VAT	visceral adipose tissue
VLDLs	very low-density lipoproteins
VNS	vagus nerve stimulation

## References

[1] Alberti K.G., Eckel R.H., Grundy S.M., Zimmet P.Z., Cleeman J.I., Donato K.A., Fruchart J.C., James W.P., Loria C.M., Smith S.C. (2009). Harmonizing the metabolic syndrome: A joint interim statement of the International Diabetes Federation Task Force on Epidemiology and Prevention; National Heart, Lung, and Blood Institute; American Heart Association; World Heart Federation; International Atherosclerosis Society; and International Association for the Study of Obesity. Circulation.

[2] Peterseim C.M., Jabbour K., Kamath Mulki A. (2024). Metabolic syndrome: An updated review on diagnosis and treatment for primary care clinicians. J. Prim. Care Community Health.

[3] Noubiap J.J., Nansseu J.R., Lontchi-Yimagou E., Nkeck J.R., Nyaga U.F., Ngouo A.T., Tounouga D.N., Tianyi F.L., Foka A.J., Ndoadoumgue A.L. (2022). Geographic distribution of metabolic syndrome and its components in the general adult population: A meta-analysis of global data from 28 million individuals. Diabetes Res. Clin. Pract..

[4] Noubiap J.J., Nansseu J.R., Nyaga U.F., Ndoadoumgue A.L., Ngouo A.T., Tounouga D.N., Tianyi F.L., Foka A.J., Lontchi-Yimagou E., Nkeck J.R. (2026). Worldwide trends in metabolic syndrome from 2000 to 2023: A systematic review and modelling analysis. Nat. Commun..

[5] Aidarbekova D., Sadykova K., Saruarov Y., Nurdinov N., Zhunissova M., Babayeva K., Nemetova D., Turmanbayeva A., Bekenova A., Nuskabayeva G. (2025). A longitudinal assessment of metabolic syndrome. J. Clin. Med..

[6] O’Riordan K.J., Moloney G.M., Keane L., Clarke G., Cryan J.F. (2025). The gut microbiota-immune-brain axis: Therapeutic implications. Cell Rep. Med..

[7] Chen C., Wang G.Q., Li D.D., Zhang F. (2025). Microbiota-gut-brain axis in neurodegenerative diseases: Molecular mechanisms and therapeutic targets. Mol. Biomed..

[8] Ataei P., Kalantari H., Bodnar T.S., Turner R.J. (2026). The gut–brain connection: Microbes’ influence on mental health and psychological disorders. Front. Microbiomes.

[9] You M., Chen N., Yang Y., Cheng L., He H., Cai Y., Liu Y., Liu H., Hong G. (2024). The gut microbiota-brain axis in neurological disorders. MedComm.

[10] Xu J., Lu Y. (2025). The microbiota-gut-brain axis and central nervous system diseases: From mechanisms of pathogenesis to therapeutic strategies. Front. Microbiol..

[11] Pan D., Li J., Chen S., Gu S., Jiang M., Xu Q. (2025). Microbiota-gut-brain axis pathogenesis and targeted therapeutics in sleep disorders. Front. Neurol..

[12] Barbu A.C., Stoleru S., Zugravu A., Poenaru E., Dragomir A., Costescu M., Aurelian S.M., Shhab Y., Stoleru C.M., Coman O.A. (2026). Dopamine and the gut microbiota: Interactions within the microbiota–gut–brain axis and therapeutic perspectives. Int. J. Mol. Sci..

[13] Doenyas C., Clarke G., Cserjési R. (2025). Gut–brain axis and neuropsychiatric health: Recent advances. Sci. Rep..

[14] Suarez B., Álvarez A.M., Mascardi M.F., Ramos A.L.M., Woo D.H., Gutiérrez M.M., Alzueta G., Basbus M.D.C., Bruzone S., Cuart P. (2026). Interactions between the gut microbiome and genetic and clinical risk factors for metabolic dysfunction-associated steatotic liver disease (MASLD) in patients with type 2 diabetes mellitus from different geographical regions of Argentina. Life.

[15] Plaza-Diaz J., Herrera-Quintana L., Olivares-Arancibia J., Vázquez-Lorente H. (2026). Personalized nutrition through the gut microbiome in metabolic syndrome and related comorbidities. Nutrients.

[16] Ayivi-Tosuh S.M., Dofuor A.K., Yamoah J.A.A., Gayi B.K., Aiduenu A.F., Akomea A., Anovunga S.A., Ekloh W., Basing L.A. (2026). Gut-microbiome interactions: Characterization, therapeutic implications and machine learning. Sage Open Pathol..

[17] Jyoti, Dey P. (2025). Mechanisms and implications of the gut microbial modulation of intestinal metabolic processes. npj Metab. Health Dis..

[18] Van Hul M., Cani P.D. (2026). From microbiome to metabolism: Bridging a two-decade translational gap. Cell Metab..

[19] Sasidharan Pillai S., Ashraf A.P. (2026). Gut microbiota and metabolic health: From dysbiosis to therapeutics. Diabetes Ther..

[20] Eckel R.H., Grundy S.M., Zimmet P.Z. (2005). The metabolic syndrome. Lancet.

[21] Petersen M.C., Shulman G.I. (2018). Mechanisms of insulin action and insulin resistance. Physiol. Rev..

[22] Taniguchi C.M., Emanuelli B., Kahn C.R. (2006). Critical nodes in signalling pathways: Insights into insulin action. Nat. Rev. Mol. Cell Biol..

[23] Hotamisligil G. (2006). Inflammation and metabolic disorders. Nature.

[24] Shoelson S.E., Lee J., Goldfine A.B. (2006). Inflammation and insulin resistance. J. Clin. Investig..

[25] Samuel V.T., Shulman G.I. (2012). Mechanisms for insulin resistance: Common threads and missing links. Cell.

[26] Saltiel A.R., Kahn C.R. (2001). Insulin signalling and the regulation of glucose and lipid metabolism. Nature.

[27] Kahn B.B., Flier J.S. (2000). Obesity and insulin resistance. J. Clin. Investig..

[28] Lowell B.B., Shulman G.I. (2005). Mitochondrial dysfunction and type 2 diabetes. Science.

[29] Cani P.D., Jordan B.F. (2018). Gut microbiota-mediated inflammation in obesity: A link with gastrointestinal cancer. Nat. Rev. Gastroenterol. Hepatol..

[30] Ji H., Su S., Chen M., Liu S., Liu S., Guo J. (2025). The role of gut microbiota in insulin resistance: Recent progress. Front. Microbiol..

[31] Chimenti I., Cammisotto V. (2024). Special Issue “Effects of dyslipidemia and metabolic syndrome on cardiac and vascular dysfunction”. Int. J. Mol. Sci..

[32] Bays H.E., Kirkpatrick C.F., Maki K.C., Toth P.P., Morgan R.T., Tondt J., Christensen S.M., Dixon D.L., Jacobson T.A. (2024). Obesity, dyslipidemia, and cardiovascular disease: A joint expert review from the Obesity Medicine Association and the National Lipid Association 2024. J. Clin. Lipidol..

[33] Dhondge R.H., Agrawal S., Patil R., Kadu A., Kothari M. (2024). A comprehensive review of metabolic syndrome and its role in cardiovascular disease and type 2 diabetes mellitus: Mechanisms, risk factors, and management. Cureus.

[34] Hamooya B.M., Siame L., Muchaili L., Masenga S.K., Kirabo A. (2025). Metabolic syndrome: Epidemiology, mechanisms, and current therapeutic approaches. Front. Nutr..

[35] Blumenthal R.S., Morris P.B., Gaudino M., Johnson H.M., Anderson T.S., Bittner V.A., Blankstein R., Brewer L.C., Cho L., Writing Committee Members (2026). 2026 ACC/AHA/AACVPR/ABC/ACPM/ADA/AGS/AphA/ASPC/NLA/PCNA. Guideline on the management of dyslipidemia: A report of the American College of Cadiology/American Heart Association Joint Committee on Clinical Practice Guidelines. Circulation.

[36] Młynarska E., Bojdo K., Bulicz A., Frankenstein H., Gąsior M., Kustosik N., Rysz J., Franczyk B. (2025). Obesity as a multifactorial chronic disease: Molecular mechanisms, systemic impact, and emerging digital interventions. Curr. Issues Mol. Biol..

[37] Després J.P. (2012). Body fat distribution and risk of cardiovascular disease: An update. Circulation.

[38] Kadowaki T., Yamauchi T. (2005). Adiponectin and adiponectin receptors. Endocr. Rev..

[39] Trayhurn P. (2013). Hypoxia and adipose tissue function and dysfunction in obesity. Physiol. Rev..

[40] Lumeng C.N., Saltiel A.R. (2011). Inflammatory links between obesity and metabolic disease. J. Clin. Investig..

[41] Friedman J.M., Halaas J.L. (1998). Leptin and the regulation of body weight in mammals. Nature.

[42] Nielsen S., Guo Z., Johnson C.M., Hensrud D.D., Jensen M.D. (2004). Splanchnic lipolysis in human obesity. J. Clin. Investig..

[43] Chandrasekaran P., Weiskirchen R. (2024). Cellular and molecular mechanisms of insulin resistance. Curr. Tissue Microenviron. Rep..

[44] DeFronzo R.A., Tripathy D. (2009). Skeletal muscle insulin resistance is the primary defect in type 2 diabetes. Diabetes Care.

[45] Roush G.C. (2019). Obesity-induced hypertension: Heavy on the accelerator. J. Am. Heart Assoc..

[46] Ouchi N., Parker J.L., Lugus J.J., Walsh K. (2011). Adipokines in inflammation and metabolic disease. Nat. Rev. Immunol..

[47] Drucker D.J. (2018). Mechanisms of action and therapeutic application of glucagon-like peptide-1. Cell Metab..

[48] Weir G.C., Bonner-Weir S. (2004). Five stages of evolving beta-cell dysfunction during progression to diabetes. Diabetes.

[49] Unger R.H., Cherrington A.D. (2012). Glucagonocentric restructuring of diabetes: A pathophysiologic and therapeutic makeover. J. Clin. Investig..

[50] Pasquali R., Vicennati V., Cacciari M., Pagotto U. (2006). The hypothalamic-pituitary-adrenal axis activity in obesity and the metabolic syndrome. Ann. N. Y. Acad. Sci..

[51] Reynolds R.M. (2013). Glucocorticoid excess and the developmental origins of disease: Two decades of testing the hypothesis—2012 Curt Richter Award Winner. Psychoneuroendocrinology.

[52] Morton N.M., Paterson J.M., Masuzaki H., Holmes M.C., Staels B., Fievet C., Walker B.R., Flier J.S., Mullins J.J., Seckl J.R. (2004). Novel adipose tissue-mediated resistance to diet-induced visceral obesity in 11 beta-hydroxysteroid dehydrogenase type 1-deficient mice. Diabetes.

[53] Dallman M.F., Pecoraro N., Akana S.F., La Fleur S.E., Gomez F., Houshyar H., Bell M.E., Bhatnagar S., Laugero K.D., Manalo S. (2003). Chronic stress and obesity: A new view of “comfort food”. Proc. Natl. Acad. Sci. USA.

[54] Rusch J.A., Layden B.T., Dugas L.R. (2023). Signalling cognition: The gut microbiota and hypothalamic-pituitary-adrenal axis. Front. Endocrinol..

[55] Grossmann M. (2011). Low testosterone in men with type 2 diabetes: Significance and treatment. J. Clin. Endocrinol. Metab..

[56] Kelly D.M., Jones T.H. (2015). Testosterone and obesity. Obes. Rev..

[57] Azziz R., Carmina E., Chen Z., Dunaif A., Laven J.S., Legro R.S., Lizneva D., Natterson-Horowtiz B., Teede H.J., Yildiz B.O. (2016). Polycystic ovary syndrome. Nat. Rev. Dis. Prim..

[58] Mauvais-Jarvis F. (2015). Sex differences in metabolic homeostasis, diabetes, and obesity. Biol. Sex Differ..

[59] Holst J.J. (2019). From the incretin concept and the discovery of GLP-1 to today’s diabetes therapy. Front. Endocrinol..

[60] Abildinova G.Z., Benberin V.V., Vochshenkova T.A., Afshar A., Mussin N.M., Kaliyev A.A., Zhussupova Z., Tamadon A. (2024). The gut-brain-metabolic axis: Exploring the role of microbiota in insulin resistance and cognitive function. Front. Microbiol..

[61] Tilg H., Moschen A.R. (2006). Adipocytokines: Mediators linking adipose tissue, inflammation and immunity. Nat. Rev. Immunol..

[62] Shoelson S.E., Herrero L., Naaz A. (2007). Obesity, inflammation, and insulin resistance. Gastroenterology.

[63] Guilherme A., Virbasius J.V., Puri V., Czech M.P. (2008). Adipocyte dysfunctions linking obesity to insulin resistance and type 2 diabetes. Nat. Rev. Mol. Cell Biol..

[64] Cani P.D., Amar J., Iglesias M.A., Poggi M., Knauf C., Bastelica D., Neyrinck A.M., Fava F., Tuohy K.M., Chabo C. (2007). Metabolic endotoxemia initiates obesity and insulin resistance. Diabetes.

[65] Montezano A.C., Touyz R.M. (2012). Molecular mechanisms of hypertension—Reactive oxygen species and antioxidants: A basic science update for the clinician. Can. J. Cardiol..

[66] Shabani M., Ghoshehy A., Mottaghi A.M., Chegini Z., Kerami A., Shariati A., Moghadam M.T. (2025). The relationship between gut microbiome and human diseases: Mechanisms, predisposing factors and potential intervention. Front. Cell. Infect. Microbiol..

[67] Rinninella E., Raoul P., Cintoni M., Franceschi F., Miggiano G.A.D., Gasbarrini A., Mele M.C. (2019). What is the healthy gut microbiota composition? A changing ecosystem across age, environment, diet, and diseases. Microorganisms.

[68] Arumugam M., Raes J., Pelletier E., Le Paslier D., Yamada T., Mende D.R., Fernandes G.R., Tap J., Bruls T., Batto J.M. (2011). Enterotypes of the human gut microbiome. Nature.

[69] Asnicar F., Berry S.E., Valdes A.M., Nguyen L.H., Piccinno G., Drew D.A., Leeming E., Gibson R., Le Roy C., Khatib H.A. (2021). Microbiome connections with host metabolism and habitual diet from 1,098 deeply phenotyped individuals. Nat. Med..

[70] Koh A., De Vadder F., Kovatcheva-Datchary P., Bäckhed F. (2016). From dietary fiber to host physiology: Short-chain fatty acids as key bacterial metabolites. Cell.

[71] Blaak E.E., Canfora E.E., Theis S., Frost G., Groen A.K., Mithieux G., Nauta A., Scott K., Stahl B., van Harsselaar J. (2020). Short chain fatty acids in human gut and metabolic health. Benef. Microbes.

[72] Neis E.P., Dejong C.H., Rensen S.S. (2015). The role of microbial amino acid metabolism in host metabolism. Nutrients.

[73] Canfora E.E., Meex R.C.R., Venema K., Blaak E.E. (2019). Gut microbial metabolites in obesity, NAFLD and T2DM. Nat. Rev. Endocrinol..

[74] Wahlström A., Sayin S.I., Marschall H.U., Bäckhed F. (2016). Intestinal crosstalk between bile acids and microbiota and its impact on host metabolism. Cell Metab..

[75] Collins S.L., Stine J.G., Bisanz J.E., Okafor C.D., Patterson A.D. (2023). Bile acids and the gut microbiota: Metabolic interactions and impacts on disease. Nat. Rev. Microbiol..

[76] Wang Z., Klipfell E., Bennett B., Koeth R., Levison B.S., Dugar B., Feldstein A.E., Britt E.B., Fu X., Chung Y.M. (2011). Gut flora metabolism of phosphatidylcholine promotes cardiovascular disease. Nature.

[77] Janeiro M.H., Ramírez M.J., Milagro F.I., Martínez J.A., Solas M. (2018). Implication of trimethylamine N-oxide (TMAO) in disease: Potential biomarker or new therapeutic target. Nutrients.

[78] Belkaid Y., Hand T.W. (2014). Role of the microbiota in immunity and inflammation. Cell.

[79] Thaiss C.A., Zmora N., Levy M., Elinav E. (2016). The microbiome and innate immunity. Nature.

[80] Depommier C., Everard A., Druart C., Plovier H., Van Hul M., Vieira-Silva S., Falony G., Raes J., Maiter D., Delzenne N.M. (2019). Supplementation with Akkermansia muciniphila in overweight and obese human volunteers: A proof-of-concept exploratory study. Nat. Med..

[81] Dalile B., Van Oudenhove L., Vervliet B., Verbeke K. (2019). The role of short-chain fatty acids in microbiota-gut-brain communication. Nat. Rev. Gastroenterol. Hepatol..

[82] Yang W., Yu T., Huang X., Bilotta A.J., Xu L., Lu Y., Sun J., Pan F., Zhou J., Zhang W. (2020). Intestinal microbiota-derived short-chain fatty acids regulation of immune cell IL-22 production and gut immunity. Nat. Commun..

[83] Zinöcker M.K., Lindseth I.A. (2018). The western diet–microbiome-host interaction and its role in metabolic disease. Nutrients.

[84] Loh J.S., Mak W.Q., Tan L.K.S., Ng C.X., Chan H.H., Yeow S.H., Foo J.B., Ong Y.S., How C.W., Khaw K.Y. (2024). Microbiota-gut-brain axis and its therapeutic applications in neurodegenerative diseases. Signal Transduct. Target. Ther..

[85] Wei L., Singh R., Ghoshal U.C. (2022). Enterochromaffin cells-gut microbiota crosstalk: Underpinning the symptoms, pathogenesis, and pharmacotherapy in disorders of gut-brain interaction. J. Neurogastroenterol. Motil..

[86] Liu L., Huh J.R., Shah K. (2022). Microbiota and the gut-brain-axis: Implications for new therapeutic design in the CNS. eBioMedicine.

[87] Li D., Lu Y., Yuan S., Cai X., He Y., Chen J., Wu Q., He D., Fang A., Bo Y. (2022). Gut microbiota-derived metabolite trimethylamine-N-oxide and multiple health outcomes: An umbrella review and updated meta-analysis. Am. J. Clin. Nutr..

[88] Devkota S., Wang Y., Musch M.W., Leone V., Fehlner-Peach H., Nadimpalli A., Antonopoulos D.A., Jabri B., Chang E.B. (2012). Dietary-fat-induced taurocholic acid promotes pathobiont expansion and colitis in Il10^−/−^ mice. Nature.

[89] Rohr M.W., Narasimhulu C.A., Rudeski-Rohr T.A., Parthasarathy S. (2020). Negative effects of a high-fat diet on intestinal permeability: A review. Adv. Nutr..

[90] Saltiel A.R., Olefsky J.M. (2017). Inflammatory mechanisms linking obesity and metabolic disease. J. Clin. Investig..

[91] Lorsch Z.S., Liddle R.A. (2026). Mechanisms and clinical implications of gut-brain interactions. J. Clin. Investig..

[92] Wolfson R.L., Abdelaziz A., Rankin G., Kushner S., Qi L., Mazor O., Choi S., Sharma N., Ginty D.D. (2023). DRG afferents that mediate physiologic and pathologic mechanosensation from the distal colon. Cell.

[93] Ravisankar P., Wong D.S.H., Zeng M.Y. (2026). Microbiota regulation of gut-brain neuroimmune crosstalk in early life. Int. Immunol..

[94] Zheng B., Shen X., Han N., Guo X., Wan S. (2026). The microbiota-gut-brain-epigenome axis as a novel therapeutic target for decoding postpartum depression. Front. Med..

[95] Rowland I., Gibson G., Heinken A., Scott K., Swann J., Thiele I., Tuohy K. (2018). Gut microbiota functions: Metabolism of nutrients and other food components. Eur. J. Nutr..

[96] Qin Y., Wade P.A. (2018). Crosstalk between the microbiome and epigenome: Messages from bugs. J. Biochem..

[97] Kurilshikov A., Medina-Gomez C., Bacigalupe R., Radjabzadeh D., Wang J., Demirkan A., Le Roy C.I., Raygoza Garay J.A., Finnicum C.T., Liu X. (2021). Large-scale association analyses identify host factors influencing human gut microbiome composition. Nat. Genet..

[98] Waters J.L., Ley R.E. (2019). The human gut bacteria Christensenellaceae are widespread, heritable, and associated with health. BMC Biol..

[99] Cardona F., Andrés-Lacueva C., Tulipani S., Tinahones F.J., Queipo-Ortuño M.I. (2013). Benefits of polyphenols on gut microbiota and implications in human health. J. Nutr. Biochem..

[100] Tang W.H., Kitai T., Hazen S.L. (2017). Gut microbiota in cardiovascular health and disease. Circ. Res..

[101] Zarrinpar A., Chaix A., Panda S. (2016). Daily eating patterns and their impact on health and disease. Trends Endocrinol. Metab..

[102] Dethlefsen L., Relman D.A. (2011). Incomplete recovery and individualized responses of the human distal gut microbiota to repeated antibiotic perturbation. Proc. Natl. Acad. Sci. USA.

[103] Blaser M.J. (2016). Antibiotic use and its consequences for the normal microbiome. Science.

[104] Becattini S., Taur Y., Pamer E.G. (2016). Antibiotic-induced changes in the intestinal microbiota and disease. Trends Mol. Med..

[105] Clauss M., Gérard P., Mosca A., Leclerc M. (2021). Interplay between exercise and gut microbiome in the context of human health and performance. Front. Nutr..

[106] Allen J.M., Mailing L.J., Niemiro G.M., Moore R., Cook M.D., White B.A., Holscher H.D., Woods J.A. (2018). Exercise alters gut microbiota composition and function in lean and obese humans. Med. Sci. Sports Exerc..

[107] Voigt R.M., Forsyth C.B., Green S.J., Engen P.A., Keshavarzian A. (2016). Circadian rhythm and the gut microbiome. Int. Rev. Neurobiol..

[108] Foster J.A., McVey Neufeld K.A. (2013). Gut-brain axis: How the microbiome influences anxiety and depression. Trends Neurosci..

[109] Biedermann L., Brülisauer K., Zeitz J., Frei P., Scharl M., Vavricka S.R., Fried M., Loessner M.J., Rogler G., Schuppler M. (2014). Smoking cessation alters intestinal microbiota: Insights from quantitative investigations on human fecal samples using FISH. Inflamm. Bowel Dis..

[110] Bajaj J.S. (2019). Alcohol, liver disease and the gut microbiota. Nat. Rev. Gastroenterol. Hepatol..

[111] Kim J. (2025). Metabotype risk clustering based on metabolic disease biomarkers and its association with metabolic syndrome in korean adults: Findings from the 2016–2023 Korea National Health and Nutrition Examination Survey (KNHANES). Diseases.

[112] Sun K., Liu J., Ning G. (2012). Active smoking and risk of metabolic syndrome: A meta-analysis of prospective studies. PLoS ONE.

[113] Schwingshackl L., Schwedhelm C., Hoffmann G., Knüppel S., Iqbal K., Andriolo V., Bechthold A., Schlesinger S., Boeing H. (2017). Food groups and risk of hypertension: A systematic review and dose-response meta-analysis of prospective studies. Adv. Nutr..

[114] Powell K.E., King A.C., Buchner D.M., Campbell W.W., DiPietro L., Erickson K.I., Hillman C.H., Jakicic J.M., Janz K.F., Katzmarzyk P.T. (2018). The scientific foundation for the physical activity guidelines for Americans, 2nd edition. J. Phys. Act. Health.

[115] Hou K., Wu Z.X., Chen X.Y., Wang J.Q., Zhang D., Xiao C., Zhu D., Koya J.B., Wei L., Li J. (2022). Microbiota in health and diseases. Signal Transduct. Target. Ther..

[116] Camilleri M. (2019). Leaky gut: Mechanisms, measurement and clinical implications in humans. Gut.

[117] Cryan J.F., O’Riordan K.J., Cowan C.S.M., Sandhu K.V., Bastiaanssen T.F.S., Boehme M., Codagnone M.G., Cussotto S., Fulling C., Golubeva A.V. (2019). The Microbiota-Gut-Brain Axis. Physiol. Rev..

[118] Agus A., Clément K., Sokol H. (2021). Gut microbiota-derived metabolites as central regulators in metabolic disorders. Gut.

[119] Mehta I., Juneja K., Nimmakayala T., Bansal L., Pulekar S., Duggineni D., Ghori H.K., Modi N., Younas S. (2025). Gut microbiota and mental health: A comprehensive review of gut-brain interactions in mood disorders. Cureus.

[120] Nakhal M.M., Yassin L.K., Alyaqoubi R., Saeed S., Alderei A., Alhammadi A., Alshehhi M., Almehairbi A., Al Houqani S., BaniYas S. (2024). The microbiota-gut-brain axis and neurological disorders: A comprehensive review. Life.

[121] Margolis K.G., Cryan J.F., Mayer E.A. (2021). The microbiota–gut–brain axis: From motility to mood. Gastroenterology.

[122] Faraji N., Payami B., Ebadpour N., Gorji A. (2025). Vagus nerve stimulation and gut microbiota interactions: A novel therapeutic avenue for neuropsychiatric disorders. Neurosci. Biobehav. Rev..

[123] Pan D., Jiang M., Wang Y., He J., Tang J., Liu S., Li M., Jiang X., Xu Q. (2025). Multi-omics reveals associations between the microbiota-gut-brain axis and antidepressant effects of vagus nerve stimulation. Neurobiol. Stress.

[124] Pires L., Gonzalez-Paramás A.M., Heleno S.A., Calhelha R.C. (2024). Gut microbiota as an endocrine organ: Unveiling its role in human physiology and health. Appl. Sci..

[125] Kellenberger A., Dewal R.S., de Wouters d’Oplinter A., Sichert A., Heine M., Fuh M.M., Slack E., Delessa Challa T., Wolfrum C. (2025). Antibiotic-induced gut microbiota depletion enhances glucose tolerance linked to GLP-1 signaling. Front. Endocrinol..

[126] Kuhre R.E., Wewer Albrechtsen N.J., Larsen O., Jepsen S.L., Balk-Møller E., Andersen D.B., Deacon C.F., Schoonjans K., Reimann F., Gribble F.M. (2018). Bile acids are important direct and indirect regulators of the secretion of appetite- and metabolism-regulating hormones from the gut and pancreas. Mol. Metab..

[127] Holst J.J. (2007). The physiology of glucagon-like peptide 1. Physiol. Rev..

[128] Kaelberer M.M., Buchanan K.L., Klein M.E., Barth B.B., Montoya M.M., Shen X., Bohórquez D.V. (2018). A gut-brain neural circuit for nutrient sensory transduction. Science.

[129] Lavelle A., Sokol H. (2020). Gut microbiota-derived metabolites as key actors in inflammatory bowel disease. Nat. Rev. Gastroenterol. Hepatol..

[130] Shafik A.N., Fahim V.F., Iskander F.A., Elsayegh H.A., Serag H., Sallam H.S. (2025). The role of the gut microbiome dysbiosis in metabolic dysfunction: A mini review. Healthcare.

[131] Rosas-Sánchez G.U., Germán-Ponciano L.J., Puga-Olguín A., Soto M.E.F., Medina A.Y.N., Muñoz-Carillo J.L., Rodríguez-Landa J.F., Soria-Fregozo C. (2025). Gut–brain axis in mood disorders: A narrative review of neurobiological insights and probiotic interventions. Biomedicines.

[132] Park J.C., Chang L., Kwon H.K., Im S.H. (2025). Beyond the gut: Decoding the gut-immune-brain axis in health and disease. Cell. Mol. Immunol..

[133] Warren A., Nyavor Y., Zarabian N., Mahoney A., Frame L.A. (2024). The microbiota-gut-brain-immune interface in the pathogenesis of neuroinflammatory diseases: A narrative review of the emerging literature. Front. Immunol..

[134] Coste S.C., Orășan O.H., Cozma A., Negrean V., Sitar-Tăut A.V., Filip G.A., Hangan A.C., Lucaciu R.L., Iancu M., Procopciuc L.M. (2024). Metabolic dysfunction-associated steatotic liver disease: The associations between inflammatory markers, TLR4, and cytokines IL-17A/F, and their connections to the degree of steatosis and the risk of fibrosis. Biomedicines.

[135] Zhao H., Tao L., Tang C., Cai W., Shen W. (2025). Do immune system and microbiome–gut–brain axis interactions associate with major depressive disorder?. J. Transl. Med..

[136] Oyovwi M.O., Uchechukwu G.J., Oyekanmi B.A., Odokuma E.I., Ogenma U.T. (2024). Gut microbiota and neuroinflammation: An interconnected nexus of health and neurodegenerative disease. OBM Neurobiol..

[137] Yuan C., He Y., Xie K., Feng L., Gao S., Cai L. (2023). Review of microbiota gut brain axis and innate immunity in inflammatory and infective diseases. Front. Cell. Infect. Microbiol..

[138] Nițulescu I.M., Ciulei G., Cozma A., Procopciuc L.M., Orășan O.H. (2023). From innate immunity to metabolic disorder: A review of the NLRP3 inflammasome in diabetes mellitus. J. Clin. Med..

[139] Pavlov V.A., Tracey K.J. (2017). Neural regulation of immunity: Molecular mechanisms and clinical translation. Nat. Neurosci..

[140] Agus A., Planchais J., Sokol H. (2018). Gut microbiota regulation of tryptophan metabolism in health and disease. Cell Host Microbe.

[141] Rana S., Ali S., Wani H.A., Mushtaq Q.D., Sharma S., Rehman M.U. (2022). Metabolic syndrome and underlying genetic determinants—A systematic review. J. Diabetes Metab. Disord..

[142] Al-Attar S.A., Pollex R.L., Ban M.R., Young T.K., Bjerregaard P., Anand S.S., Yusuf S., Zinman B., Harris S.B., Hanley A.J. (2008). Association between the FTO rs9939609 polymorphism and the metabolic syndrome in a non-Caucasian multi-ethnic sample. Cardiovasc. Diabetol..

[143] Song Y., Wade H., Zhang B., Xu W., Wu R., Li S., Su Q. (2023). Polymorphisms of fat mass and obesity-associated gene in the pathogenesis of child and adolescent metabolic syndrome. Nutrients.

[144] Saadi H., Nagelkerke N., Carruthers S.G., Benedict S., Abdulkhalek S., Reed R., Lukic M., Nicholls M.G. (2008). Association of TCF7L2 polymorphism with diabetes mellitus, metabolic syndrome, and markers of beta cell function and insulin resistance in a population-based sample of Emirati subjects. Diabetes Res. Clin. Pract..

[145] Al-Odinan M.S., Aljefree N.M., Almoraie N.M., Bakarman M.A., Alhadrami H.A., Shatwan I.M. (2025). Interaction between the TCF7L2 gene and dietary intake on metabolic syndrome risk factors among Saudi Arabian adults. Front. Nutr..

[146] Valeeva F.V., Medvedeva M.S., Khasanova K.B., Valeeva E.V., Kiseleva T.A., Egorova E.S., Pickering C., Ahmetov I.I. (2022). Association of gene polymorphisms with body weight changes in prediabetic patients. Mol. Biol. Rep..

[147] Tang Y., Yin L., Lin F. (2024). Association of rs2241766 and rs1501299 polymorphisms in the adiponectin gene with metabolic syndrome. Immun. Inflamm. Dis..

[148] Xu C., Bai R., Zhang D., Li Z., Zhu H., Lai M., Zhu Y. (2013). Effects of APOA5 -1131T>C (rs662799) on fasting plasma lipids and risk of metabolic syndrome: Evidence from a case-control study in China and a meta-analysis. PLoS ONE.

[149] Mozafari S., Ashoori M., Emami Meybodi S.M., Solhi R., Mirjalili S.R., Firoozabadi A.D., Soltani S. (2024). Association between APOA5 polymorphisms and susceptibility to metabolic syndrome: A systematic review and meta-analysis. BMC Genom..

[150] Zhang Y., Li S., Nie H., Wang X., Li X., Wen J., Li M., Song Y. (2023). The rs17782313 polymorphism near MC4R gene confers a high risk of obesity and hyperglycemia, while PGC1α rs8192678 polymorphism is weakly correlated with glucometabolic disorder: A systematic review and meta-analysis. Front. Endocrinol..

[151] Krautkramer K.A., Fan J., Bäckhed F. (2021). Gut microbial metabolites as multi-kingdom intermediates. Nat. Rev. Microbiol..

[152] Martínez-Montoro J.I., Martín-Núñez G.M., González-Jiménez A., Garrido-Sánchez L., Moreno-Indias I., Tinahones F.J. (2024). Interactions between the gut microbiome and DNA methylation patterns in blood and visceral adipose tissue in subjects with different metabolic characteristics. J. Transl. Med..

[153] Zeevi D., Korem T., Zmora N., Israeli D., Rothschild D., Weinberger A., Ben-Yacov O., Lador D., Avnit-Sagi T., Lotan-Pompan M. (2015). Personalized nutrition by prediction of glycemic responses. Cell.

[154] Kolodziejczyk A.A., Zheng D., Elinav E. (2019). Diet-microbiota interactions and personalized nutrition. Nat. Rev. Microbiol..

[155] Goodrich J.K., Waters J.L., Poole A.C., Sutter J.L., Koren O., Blekhman R., Beaumont M., Van Treuren W., Knight R., Bell J.T. (2014). Human genetics shape the gut microbiome. Cell.

[156] Hall A.B., Tolonen A.C., Xavier R.J. (2017). Human genetic variation and the gut microbiome in disease. Nat. Rev. Genet..

[157] Knights D., Silverberg M.S., Weersma R.K., Gevers D., Dijkstra G., Huang H., Tyler A.D., van Sommeren S., Imhann F., Stempak J.M. (2014). Complex host genetics influence the microbiome in inflammatory bowel disease. Genome Med..

[158] Rausch P., Rehman A., Künzel S., Häsler R., Ott S.J., Schreiber S., Rosenstiel P., Franke A., Baines J.F. (2011). Colonic mucosa-associated microbiota is influenced by an interaction of Crohn disease and FUT2 (Secretor) genotype. Proc. Natl. Acad. Sci. USA.

[159] Johansson M.E., Hansson G.C. (2016). Immunological aspects of intestinal mucus and mucins. Nat. Rev. Immunol..

[160] Goodrich J.K., Davenport E.R., Beaumont M., Jackson M.A., Knight R., Ober C., Spector T.D., Bell J.T., Clark A.G., Ley R.E. (2016). Genetic determinants of the gut microbiome in UK twins. Cell Host Microbe.

[161] Turnbaugh P.J., Ley R.E., Mahowald M.A., Magrini V., Mardis E.R., Gordon J.I. (2006). An obesity-associated gut microbiome with increased capacity for energy harvest. Nature.

[162] Goldberg A.D., Allis C.D., Bernstein E. (2007). Epigenetics: A landscape takes shape. Cell.

[163] Ling C., Rönn T. (2019). Epigenetics in human obesity and type 2 diabetes. Cell Metab..

[164] Barres R., Zierath J.R. (2011). DNA methylation in metabolic disorders. Am. J. Clin. Nutr..

[165] Nilsson E., Ling C. (2017). DNA methylation links genetics, fetal environment, and an unhealthy lifestyle to the development of type 2 diabetes. Clin. Epigenet..

[166] Kirchner H., Osler M.E., Krook A., Zierath J.R. (2013). Epigenetic flexibility in metabolic regulation: Disease cause and prevention?. Trends Cell Biol..

[167] Sandovici I., Morais T., Constância M., Monteiro M.P. (2025). Epigenetic changes associated with obesity-related metabolic comorbidities. J. Endocr. Soc..

[168] Anderson O.S., Sant K.E., Dolinoy D.C. (2012). Nutrition and epigenetics: An interplay of dietary methyl donors, one-carbon metabolism and DNA methylation. J. Nutr. Biochem..

[169] Barker D.J. (2004). The developmental origins of adult disease. J. Am. Coll. Nutr..

[170] Hardy T.M., Tollefsbol T.O. (2011). Epigenetic diet: Impact on the epigenome and cancer. Epigenomics.

[171] Voisin S., Eynon N., Yan X., Bishop D.J. (2015). Exercise training and DNA methylation in humans. Acta Physiol..

[172] Alegría-Torres J.A., Baccarelli A., Bollati V. (2011). Epigenetics and lifestyle. Epigenomics.

[173] Skerrett-Byrne D.A., Pepin A.S., Laurent K., Beckers J., Schneider R., Hrabě de Angelis M., Teperino R. (2025). Dad’s diet shapes the future: How paternal nutrition impacts placental development and childhood metabolic health. Mol. Nutr. Food Res..

[174] Lin X., Han H., Wang N., Wang C., Qi M., Wang J., Liu G. (2024). The gut microbial regulation of epigenetic modification from a metabolic perspective. Int. J. Mol. Sci..

[175] Fellows R., Denizot J., Stellato C., Cuomo A., Jain P., Stoyanova E., Balázsi S., Hajnády Z., Liebert A., Kazakevych J. (2018). Microbiota derived short chain fatty acids promote histone crotonylation in the colon through histone deacetylases. Nat. Commun..

[176] Bonder M.J., Kurilshikov A., Tigchelaar E.F., Mujagic Z., Imhann F., Vila A.V., Deelen P., Vatanen T., Schirmer M., Smeekens S.P. (2016). The effect of host genetics on the gut microbiome. Nat. Genet..

[177] Turpin W., Espin-Garcia O., Xu W., Silverberg M.S., Kevans D., Smith M.I., Guttman D.S., Griffiths A., Panaccione R., Otley A. (2016). Association of host genome with intestinal microbial composition in a large healthy cohort. Nat. Genet..

[178] Denou E., Lolmède K., Garidou L., Pomie C., Chabo C., Lau T.C., Fullerton M.D., Nigro G., Zakaroff-Girard A., Luche E. (2015). Defective NOD2 peptidoglycan sensing promotes diet-induced inflammation, dysbiosis, and insulin resistance. EMBO Mol. Med..

[179] Vijay-Kumar M., Aitken J.D., Carvalho F.A., Cullender T.C., Mwangi S., Srinivasan S., Sitaraman S.V., Knight R., Ley R.E., Gewirtz A.T. (2010). Metabolic syndrome and altered gut microbiota in mice lacking Toll-like receptor 5. Science.

[180] Plaza-Diaz J., Ruiz-Ojeda F.J., Gil-Campos M., Gil A. (2019). Mechanisms of action of probiotics. Adv. Nutr..

[181] de Groot P., Scheithauer T., Bakker G.J., Prodan A., Levin E., Khan M.T., Herrema H., Ackermans M., Serlie M.J.M., de Brauw M. (2020). Donor metabolic characteristics drive effects of faecal microbiota transplantation on recipient insulin sensitivity, energy expenditure and intestinal transit time. Gut.

[182] Fan Y., Pedersen O. (2021). Gut microbiota in human metabolic health and disease. Nat. Rev. Microbiol..

